# Analysis of the oral microbiome during hormonal cycle and its alterations in menopausal women: the “AMICA” project

**DOI:** 10.1038/s41598-022-26528-w

**Published:** 2022-12-21

**Authors:** A. Tramice, D. Paris, A. Manca, F. A. Guevara Agudelo, S. Petrosino, L. Siracusa, M. Carbone, D. Melck, F. Raymond, F. Piscitelli

**Affiliations:** 1 CNR Istituto Di Chimica Biomolecolare, Pozzuoli (NA), Italy; 2 CNR Istituto di Ricerca Genetica e Biomedica (IRGB), Sassari, Italy; 3 Université Laval, Québéc City, Canada; 4 Epitech Group SpA, Saccolongo (PD), Italy

**Keywords:** Microbiome, Metabolomics, Lipidomics, Glycosides

## Abstract

The maintenance of human health is dependent on a symbiotic relationship between humans and associated bacteria. The diversity and abundance of each habitat’s signature microbes vary widely among body areas and among them the oral microbiome plays a key role. Significant changes in the oral cavity, predominantly at salivary and periodontal level, have been associated with changes in estrogen levels. However, whether the oral microbiome is affected by hormonal level alterations is understudied. Hence the main objective pursued by AMICA project was to characterize the oral microbiome (saliva) in healthy women through: profiling studies using "omics" technologies (NMR-based metabolomics, targeted lipidomics by LC–MS, metagenomics by NGS); SinglePlex ELISA assays; glycosidase activity analyses and bioinformatic analysis. For this purpose, thirty-nine medically healthy women aged 26–77 years (19 with menstrual cycle and 20 in menopause) were recruited. Participants completed questionnaires assessing detailed medical and medication history and demographic characteristics. Plasmatic and salivary levels of sexual hormones were assessed (FSH, estradiol, LH and progesteron) at day 3 and 14 for women with menstrual cycle and only once for women in menopause. Salivary microbiome composition was assessed through meta-taxonomic 16S sequencing and overall, the salivary microbiome of most women remained relatively stable throughout the menstrual cycle and in menopause. Targeted lipidomics and untargeted metabolomics profiling were assessed through the use of LC–MS and NMR spectroscopy technologies, respectively and significant changes in terms of metabolites were identified in saliva of post-menopausal women in comparison to cycle. Moreover, glycosyl hydrolase activities were screened and showed that the β-D-hexosaminidase activity was the most present among those analyzed. Although this study has not identified significant alterations in the composition of the oral microbiome, multiomics analysis have revealed a strong correlation between 2-AG and α-mannosidase. In conclusion, the use of a multidisciplinary approach to investigate the oral microbiome of healthy women provided some indication about microbiome-derived predictive biomarkers that could be used in the future for developing new strategies to help to re-establish the correct hormonal balance in post-menopausal women.

## Introduction

Estrogen fluctuations may play a key role in microbiome modulation, even if this phenomenon is understudied in current research. Puberty, menstruation, pregnancy and menopause are all physiological conditions characteristic of women’s life in which estrogen fluctuations occur and in turn can affect microbiome. Moreover, it is worth to note that during all these phases an increase in inflammatory processes in the oral mucosa occurs^1–3^. Among these conditions, the menopause takes on an important significance. Indeed, with the prolongation of the average lifespan, women spend at least one third of their life in the post-menopause stage, a period that is vulnerable to comorbidities. The transition into the menopausal time or peri-menopause, that surround the final years of a woman’s reproductive life, is an inflammatory process that brings several physiological changes in the body^4^. Increasing evidences have showed that the transition to post-menopausal stage is associated with a chronic systemic inflammation and may accelerate ovarian failure^4^. Moreover, this transition is associated with other alterations such as decline in brain glucose metabolism and mitochondrial respiration, myelin catabolism reduction of brain white matter volume, beta-amyloid deposition in brain, and changes in neurological function as reviewed in^5^. Finally, assumption of xenoestrogens during the post-menopause and the associated inflammatory-state will exacerbate the inflammation, which in turn might compromise the BBB and induce prolonged neuroinflammation and neurological diseases^5^. For this reason, our understanding of the different stages of female lifespan needs to be improved, potentially also by using the microbiome as a targeted therapeutic strategy.

During menopause both gut and vaginal microbiota has been extensively investigated, but not the oral one. The oral microbiome represents one of the most complex and diverse human microbiomes^6–8^ and is of great importance for its implications on the health status not only of the oral cavity, but also at a systemic level. There are several specific microbial habitats in the oral cavity that include lips, teeth, gingival sulcus, tongue, cheeks, palate and tonsils. These districts appear to be colonized by different microbial species, as well as different viral and fungal species^8,9^, that are in symbiosis with the host. However, very few studies correlate alterations of the oral bacterial composition with hormonal variations of the menstrual cycle^5–7^, showing controversial results. In the study conducted by Fischer et al.^6^, the association between menstruation and levels of sub-gingival bacteria is investigated and no correlation between the periodicity of hormonal changes and changes in microbial profiles is reported. Differently, Prout and Hopps^7^ find a possible association between ovulation and the levels of anerobic bacteria in saliva, although the study is based on a very small sample number. However, in another study by Calil et al.^5^, no association is found between anerobic bacteria and the menstrual cycle. In addition, a pilot study about 20 menopausal women associates the salivary pH values in the presence or absence of oral symptoms (dry mouth, impaired taste, feeling of heat) with the number and quality of bacterial colonies present in the saliva^8^. Indeed, the majority of the studies present in literature correlates the oral microbiome in women with menstrual cycle and/or in menopause with oral infectious diseases including dental caries and periodontitis. Salivary glands and human oral epithelium have been shown to express estrogen receptor-beta^10^ and it has been demonstrated an age-related variations in the pattern of exfoliated normal buccal mucosal cells in female subjects that probably reflect fluctuations in the hormonal levels^11^. In addition, due to microscopic similarities in cellular alterations between the vaginal and buccal epithelia reflecting the hormonal state of the menstrual cycle^11^ and in postmenopausal women^12,13^, an exhaustive investigation of the impact of female sex hormones on the oral microbiota composition may be clinically relevant since many women in menopause claim oral discomforts in addition to climacteric symptoms.

In particular, a study of Meurman et al. reports that postmenopausal women suffer from dry mouth, burning mouth (glossodynia) and hyposalivation which, in turn, may increase the occurrence of oral mucosal and dental diseases, such as candidiasis^14,15^. Alterations in salivary function and composition may affect the normal homeostasis of oral health, which in turn may lead to alterations in salivary microbiome composition, with significant impact on the quality of life^16,17^. Saliva is an attractive diagnostic fluid because it has several key advantages for disease diagnosis and prognosis, including low invasiveness, minimum cost, and easy sample collection and processing. Many experimental findings have indicated a strong relationship between female sex hormones and microbiota composition in different sites of the body. For example, the onset of some autoimmune diseases, such as asthma, occurs during the women reproductive period. In mice it has been shown that there is a strong connection between female sexual hormones, microbiome and immunity in the development of autoimmune diseases, including diabetes 1 and rheumatoid arthritis^18^.

A deeper and more exhaustive salivary microbiome characterization is necessary through multidisciplinary and omics approaches according to the different hormone levels in fertile and post-fertile subjects. In this context, the AMICA project aims to implement a pipeline of analysis of the oral microbiome with a multidisciplinary approach to characterize and investigate the relationship between this and the variations in hormone levels that describe the woman's life and its health state. In particular, the main objective pursued by AMICA is to characterize the oral microbiome (saliva) in 39 healthy women (19 of childbearing age and 20 postmenopausal) through an integrative approach with profiling studies using "omics" technologies (NMR, mass spectrometry, meta-taxonomic), ELISA assays and analysis of glycosidase activities.

## Results

### Plasmatic and salivary hormones

**Figure 1:**
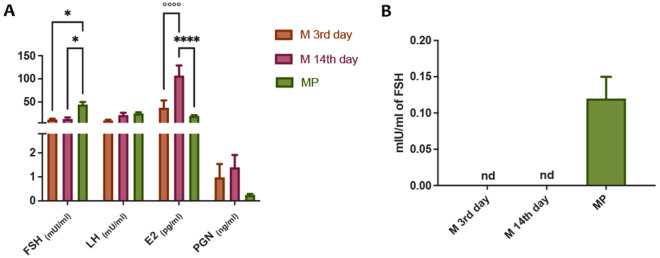
Plasmatic and salivary hormones in women with menstrual cycle at 3rd and 14th day (M 3rd day and M 14th day) and in menopause (MP). **(A)** Sex hormone levels in plasma in the three different group of women [follicle stimulating hormone (FSH), estradiol (E2), luteinizing hormone (LH), and progesterone (PGN)]. **(B)** Salivary FSH levels (not detectable, nd). ^*^ and ^°^ indicate significant differences compared to MP and M 14th day, respectively. *P* < 0.05 was considered statistically significant. Two-way ANOVA, followed by Tukey post-hoc.

Plasmatic FSH, LH, E2 and PGN were measured for women with menstrual cycle at 3rd (M-3rd day) and 14th day (M-14th day) and at one time point for post-menopausal women (MP) (Fig. 1A). As expected, E2 increased significantly in the M-14th day group as compared with M-3rd day, due to the ovulation stage, whereas the other hormones remained stable between these two groups. Moreover, FSH levels increased significantly in the MP group as compared to both M-3rd and M-14th days, whereas E2 decreased significantly in comparison with the same groups. Levels of salivary FSH is reported. Very interestingly, FSH was detectable only in MP samples and not in saliva coming from women with menstrual cycle (Fig. 1B). On the other hand, levels of other hormones did not change between the three groups (Supplementary Table S1).

### Microbiota analysis

The 16S meta-taxonomic analysis identified 72 families and 76 genera, with relative abundances varying from less than 0.1 to 85 percent; discrepancy between number of families and number of genera identified reflects on one side the belonging of several genera to the same family, on the other side the impossibility to assign some reads to a specific genus.

**Figure 2:**
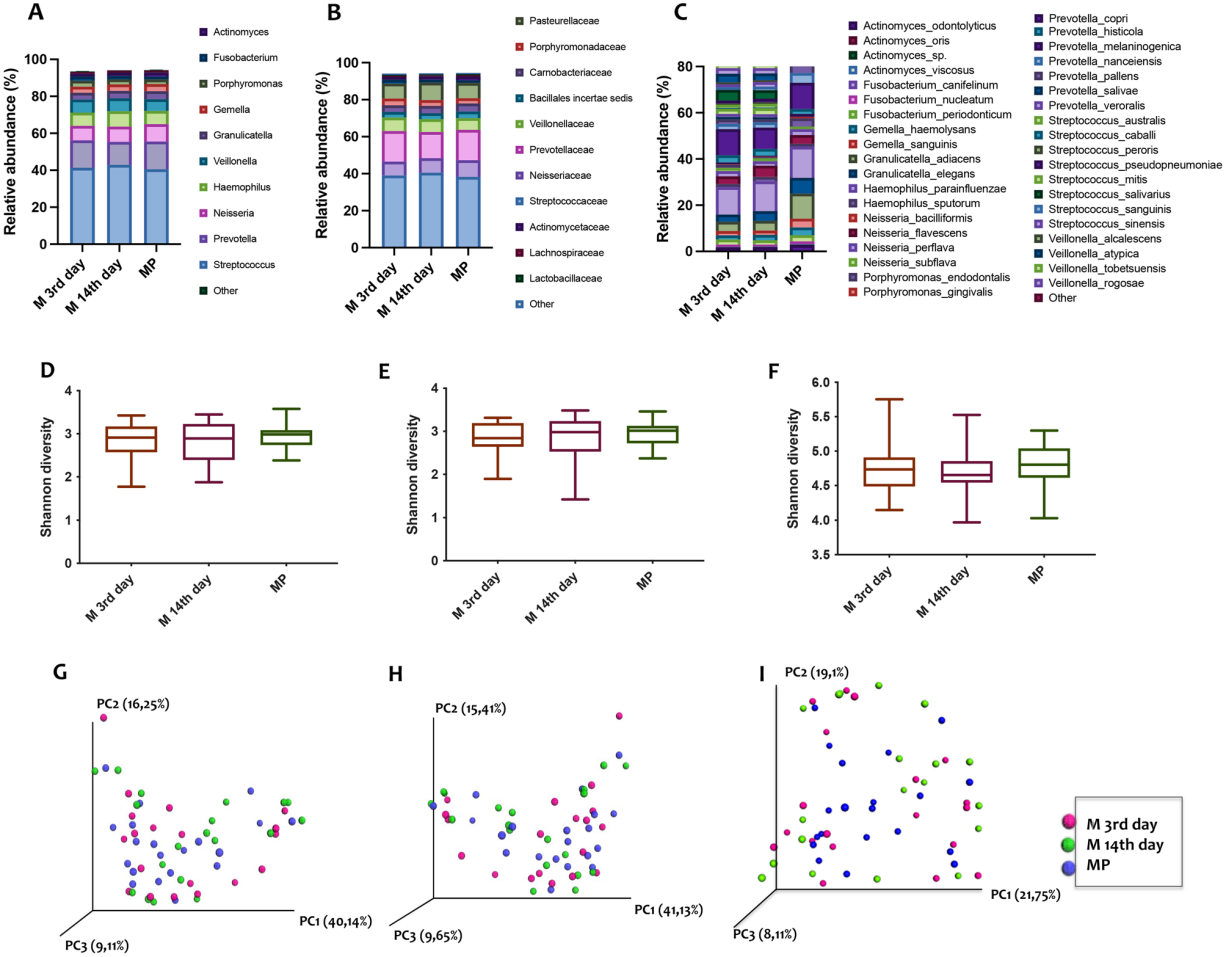
Differences in abundances of taxa in salivary samples from women with menstrual cycle at 3rd and 14th day (M-3rd day and M-14th day) and in menopause (MP) (**A**, **B**, **C**) and alpha-and beta-diversity comparisons of the gut microbiomes of each group (**D**–**I**). **(A)** Relative abundances of genera, **(B)** families and specie (**C**). (**D**-**F**) Boxplot of the Shannon diversity of each group. (**G**-**I**) Principal coordinate analysis of Bray–Curtis distances. The colors of the boxplots and dots represent the different groups analyzed according to the legend.

In particular, the genera most abundant (present in % > 1.0) were the following: *Streptococcus, Prevotella, Neisseria, Haemophilus, Veillonella, Granulicatella, Gemella, Porphyromonas, Fusobacterium, Actinomyces, and Rothia* (Fig. 2A)*.* Genera identified with relative abundance below 1% were not equally distributed in all samples. The full list is included as Supplemetary Table S2 (families) and Table S3 (genera) as mean ± SEM of the relative abundance. Raw counts for meta-taxonomic 16S microbiome profiling at the genera rank is reported as Supplementary excel file (Table S4). In Fig. 2B–C families and species are shown. Alpha (Shannon) and beta-diversity (Bray–Curtis distance) are reported in Fig. 2D–I. An OPLS-DA was applied to meta-taxonomic analysis and reported in Figure S1. The supervised regression yielded 0 components that is no latent components to drive class separation according to microbiota content expressed in the experimental conditions (the 3rd, the 14th fertile subjects and the menopause class). On the other side, unsupervised PCA produced a 5 components statistical model (R2 = 0.92, Q2 = 0.4) with no distinct cluster for the 3 groups. As we can observe from the scores plot in Figure S1A , there are some outliers and other samples which fall very far from the origin of the model plane; those microbiota content often belongs to the same subjects, regardless to the menstrual cycle phase (as for example M15, M16, M11, M17). Moreover, by exploring the associate loadings plot in Figure S1B we can deduce that the dominant families responsible for those data distribution are *Prevotella* and *Streptococcus*.

The composition of salivary microbiome in terms of both families and genera remained relatively stable throughout the menstrual cycle and menopause. Statistical analysis at the rank of species did not show significance of any taxa after FDR correction. However, two taxa could be considered to be significantly different between experimental groups before correction (Figure S2). Indeed, *Prevotella copri* was significantly higher in the MP group compared to M-14th group, and *Veillonella tobetsuensis* was significantly lower in MP group compared to the two other groups.

### Glycoside hydrolases activities

α-mannosidase, β-mannosidase, α-fucosidase, β-galactosidase, α-glucosidase, β-N-acetyl-glucosamminidase and β-glucosidase activities were screened in saliva samples.

**Figure 3:**
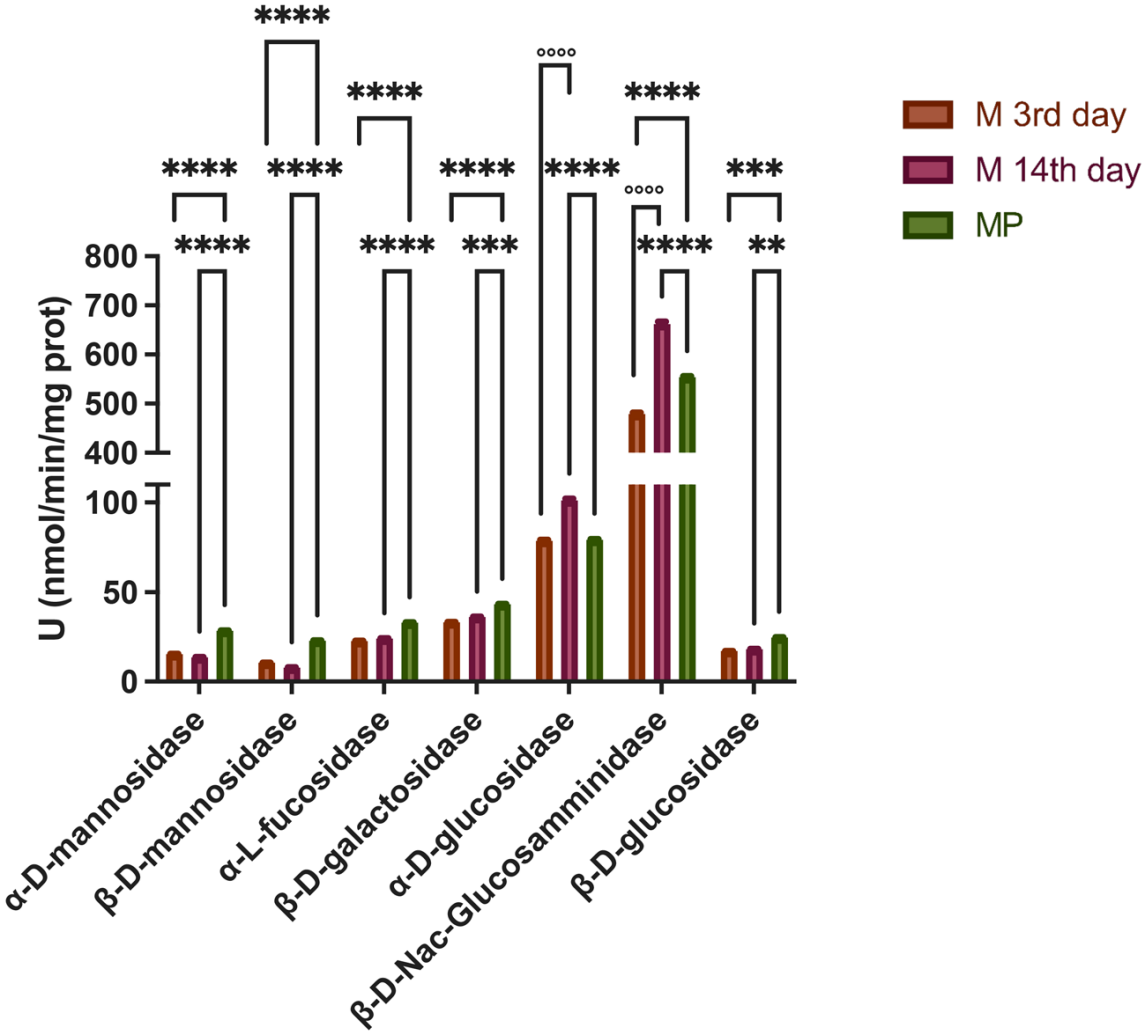
Screening of Glycosyde Hydrolases Activities in saliva. ^*^ and ^°^ indicate significant differences compared to MP and M 14th day, respectively. *P* < 0.05 was considered statistically significant. Two-way ANOVA, followed by Tukey post-hoc.

As shown in Fig. 3 almost all glycosyl hydrolase activities screened were higher in MP group (M- 3rd or M-14th day vs. MP *p* < 0,0001). However, α-glucosidase and β-N-acetyl-glucosamminidase were higher in M-14th day group as compared to M-3rd day and MP samples (*p* < 0,0001). The association between GH activities and hormone levels were evaluated through Pearson’s correlation analysis but no significance was observed (Figure S3).

### Untargeted NMR-based metabolomic analysis

**Figure 4:**
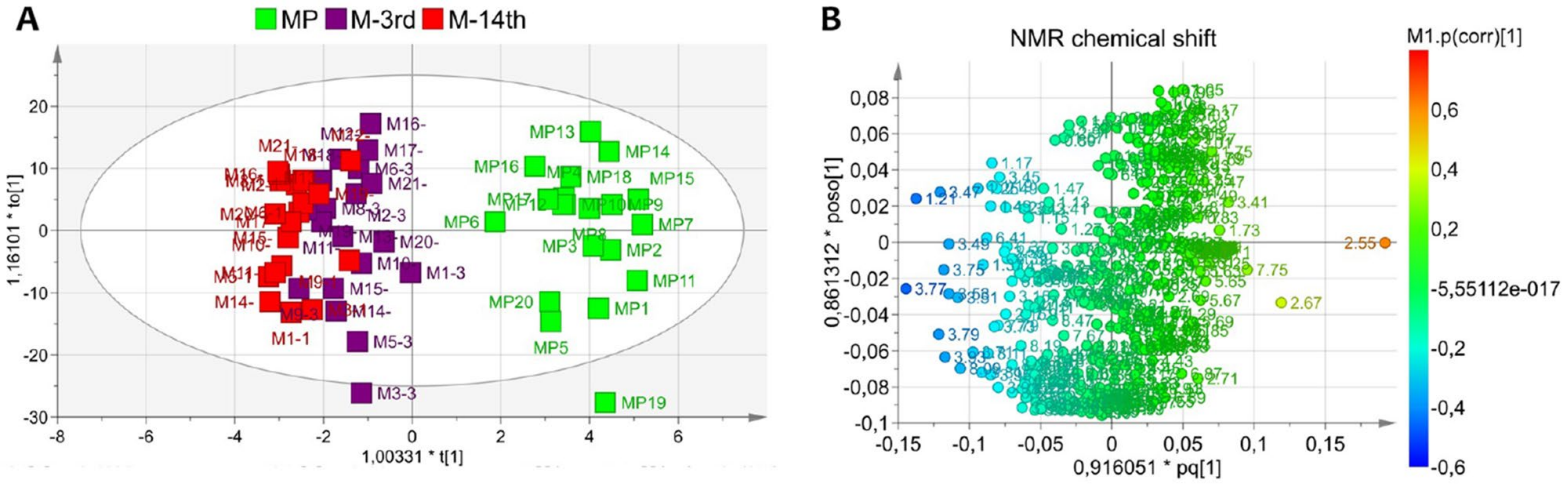
Multivariate analysis of NMR-based metabolomics data. **(A)** Orthogonal Projections to Latent Structures Discriminant Analysis (OPLS-DA) displaying data projection (menopause MP, green squares; menstrual M-3rd day and M-14th, purple and red squares, respectively) and class separation along the predictive component t[1] and **(B)** loadings plot showing NMR spectral variables responsible for saliva samples distribution.

To reveal different metabolic patterns characterizing saliva alteration during the physiological feminine life cycle from the fertile to the non-fertile period, NMR-based metabolomic analysis was conducted on saliva samples. The OPLS-DA regression resulted in a non-overfitted model (R2 = 0.51, Q2 = 0.28) with one parallel and 1 orthogonal component. The scores plot in Fig. 4A showed a clear class discrimination along the predictive component of all fertile saliva (at t[1] negative values) collected from both women at their 3rd day (M-3rd, purple squares) or the 14th day (M-14th,red squares) of the menstrual period, versus the menopausal samples (MP, green squares) placed at t[1] positive coordinates. In particular, assigning metabolites to the variables expressed in the associated loadings plot in Fig. 4B, high levels of galactose (3.47–3.49 ppm), fucose (1.25 and 3.81 ppm) and n-butyrate (1.21 ppm) were observed in all saliva acquired from fertile women, while the menopausal group exhibited high content of citrate (2.55 and 2.67 ppm), histidine (7.75 ppm), taurine (3.41 and 3.25 ppm) and polyamine (putrescine,1.73–1.75 ppm).

The correlation analysis between salivary metabolites and plasmatic hormones (Figure S4) showed no significant association.

**Figure 5:**
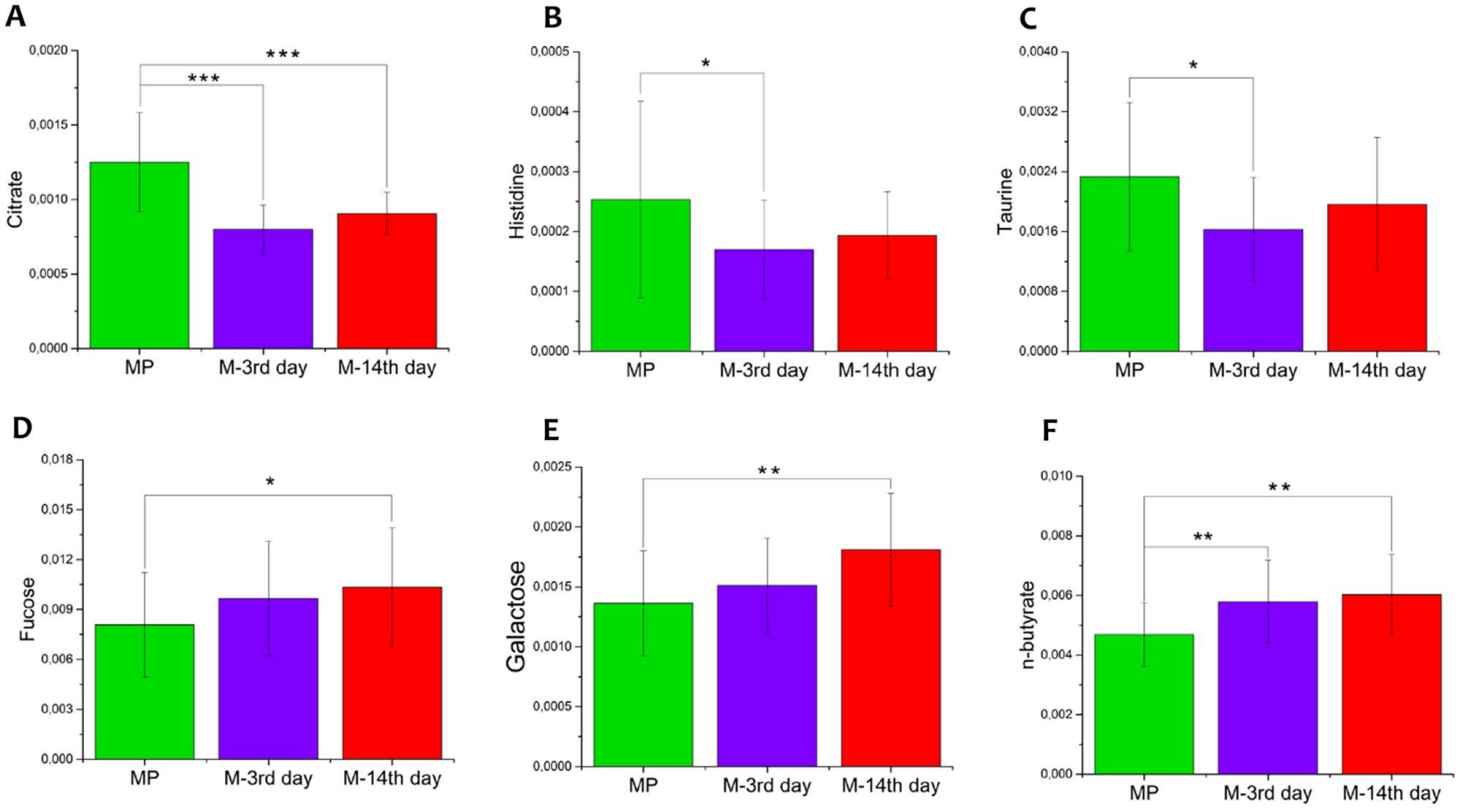
Bar plot showing normalized bin intensities of discriminant metabolites evaluated for menopause MP (green bar), menstrual M-3rd (purple bar) and M-14th (red bar) saliva classes. **(A)** Citrate, **(B)** Histidine, **(C)** Taurine, **(D)** Fucose, **(E)** Galactose and **(F)** n-Butyrate. Univariate test significance is reported as *p < 0.05 and **p < 0.001.

**Figure 6:**
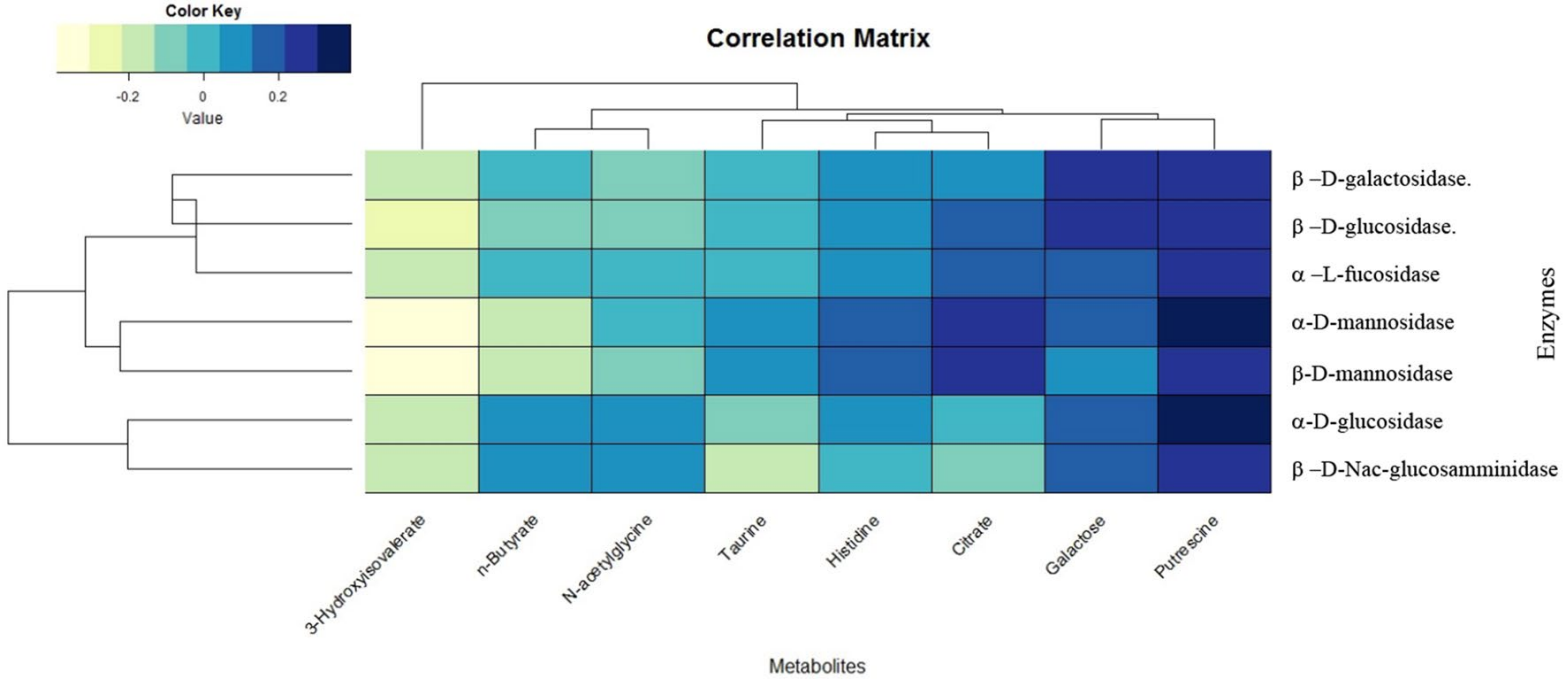
Correlation map based on Pearson correlation coefficients between selected metabolites and enzymes. Rows and columns are rearranged according to the WARD-based correlation matrix–based hierarchical clustering (CMBHC).

Bin integrations of statistically significant metabolites are reported as bars plot in Fig. 5A–F. To further associate those metabolic alterations with the glycosidase activities measured from each sample, we integrate metabolites and enzymes expressions through hierarchical analysis. The associated heatmap (Fig. 6) revealed weak positive correlations between putrescine with α-D-glucopyranoside and α-D-mannopyranoside activities, while negative correlations were found between both the short-chain fatty acids 3-hydroxyisovalerate and n-butyrate with α- and β-D-mannosidase.

Moreover, to investigate metabolites variation among the same subjects when analyzing salivary samples collected at different time points of the menstrual cycle (3rd and 14th day), we adopted a multilevel approach able to reveal metabolomic changes excluding individual factors, so to emphasize only those molecular variations depending on the hormonal status (Figure S5). Multilevel PLS-DA was applied to the reduced spectral dataset using mixOmics routine in R^19^. A 2 component regression resulted in a total of 18% of data variation explained without a discrimination of samples according to the menstrual cycle condition. In particular, the projection of all samples in the scores plot (Figure S5A) is contained within the confident ellipses delimiting the two classes (confidence level set to 95%). The variables mainly characterizing the total saliva distribution at high values along the component1 in the correlation circle plot (Figure S1B) are glucose (5.23, 3.75 and 3.77), acetate (1.93) and valine (2.25). On the other side, variables characterizing the distribution at negative coordinates are mostly related to polyamine (1.83–1.87), aspartate (2.85–2.87). None of those metabolites were found discriminant for the whole dataset analysis.

Moreover, to investigate the impact of hormonal alteration on female metabolic pathways and networks, we applied enrichment metabolic analysis, which provides mechanistic insight into the underlying biology of differentially expressed metabolites between fertile and non-fertile women. The algorithm found a network with 150 nodes with a threshold of* p *< 0.01, which are reported in Figure S6 and Table S5 NMR of the Supporting Information. In particular, the pathways enriched are the following:

- Citrate cycle (TCA cycle);
- Galactose metabolism;
- Valine, leucine and isoleucine degradation;
- Arginine and proline metabolism;
- Taurine and hypotaurine metabolism;
- Glycosphingolipid biosynthesis—ganglio series;
- Lysosome;
- Taste transduction;
- Glucagon signaling pathway;
- Type I diabetes mellitus;
- Carbohydrate digestion and absorption;
- Protein digestion and absorption;
- Central carbon metabolism in cancer.

### Targeted lipidomics analysis

**Figure 7:**
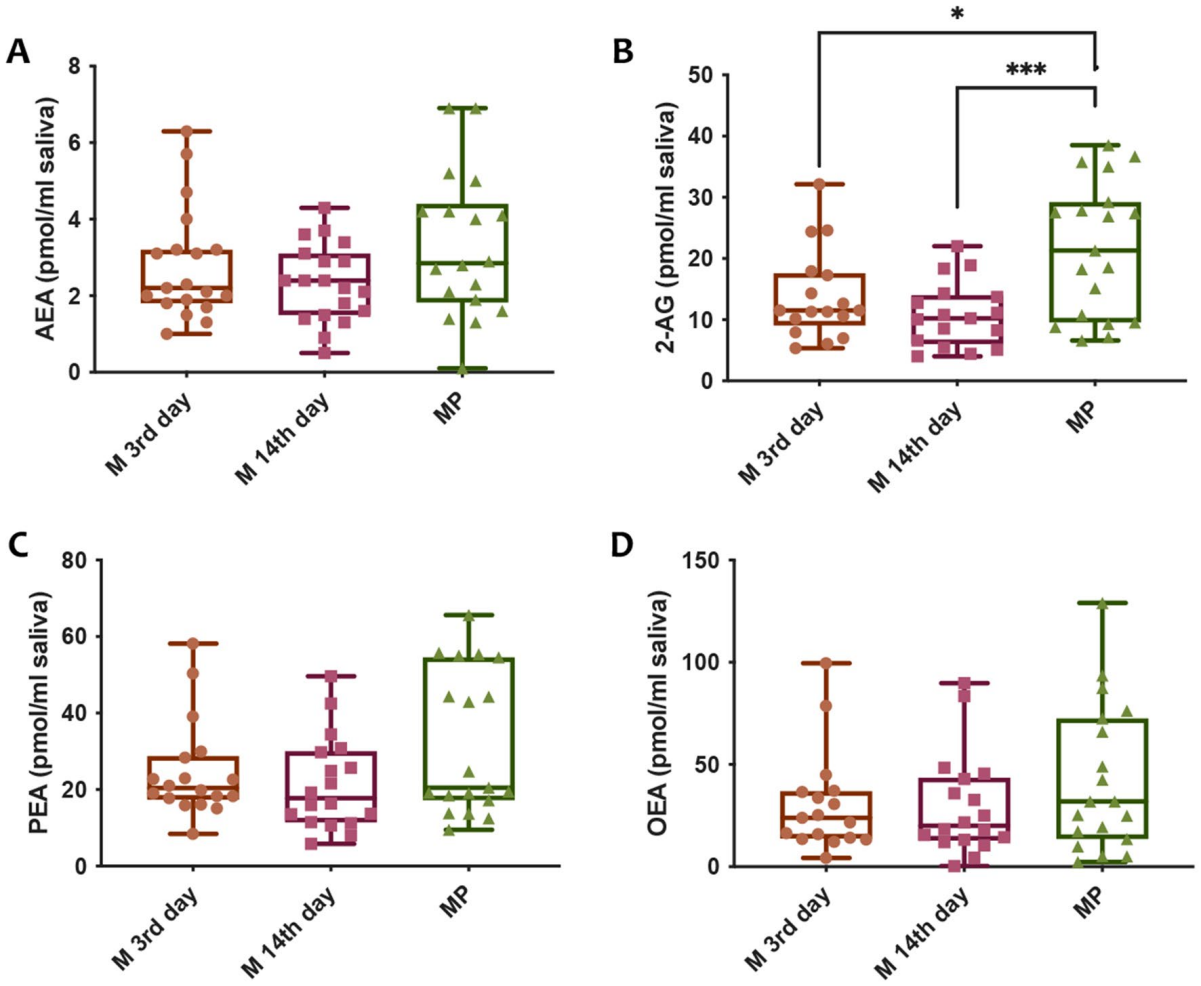
Targeted lipidomic analysis of salivary samples. **(A)** Anandamide (*N* arachidonoylethanolamine, AEA) levels expressed in pmol/ml of saliva; **(B)** 2-arachidonoylglycerol (2-AG) levels expressed in pmol/ml of saliva; **(C)** palmitoylethanolamide (PEA) levels expressed in pmol/ml of saliva and **(D)** oleoylethanolamide (OEA) levels expressed in pmol/ml of saliva. ^*^ and ^°^ indicate significant differences compared to MP and M-14th day, respectively. *P* < 0.05 was considered statistically significant. One-way ANOVA, followed by Tukey post-hoc.

Since increasing evidences in the last decade have shown a connection between the endocannabinoid (eCB) system and the microbiome, especially in the gut and in the gut-brain axis, as reviewed elsewhere^20–23^, and it is well known the important role of the eCBs in the reproduction and in the interplay with estrogen system^24^, a targeted lipidomics approach was applied to identify and quantify the main eCBs and *N*-acylethanolamine related in saliva samples. As shown in Fig. 7, the anandamide (*N*-arachidonoylethanolamine, AEA, Fig. 7A), 2-arachidonoylglycerol (2-AG, Fig. 7B), palmitoylethanolamide (PEA, Fig. 7C) and oleoylethanolamide (OEA, Fig. 7D) were quantified. 2-AG levels were significantly higher in saliva of menopausal women than in saliva from fertile women and this could be correlated with hormones levels (Fig. 7B). Moreover, we analyzed the correlation between hormone levels and eCBs (Figure S7) but Pearson’s correlation analysis did not show any significant association.

### Multi-omics analysis

**Figure 8:**
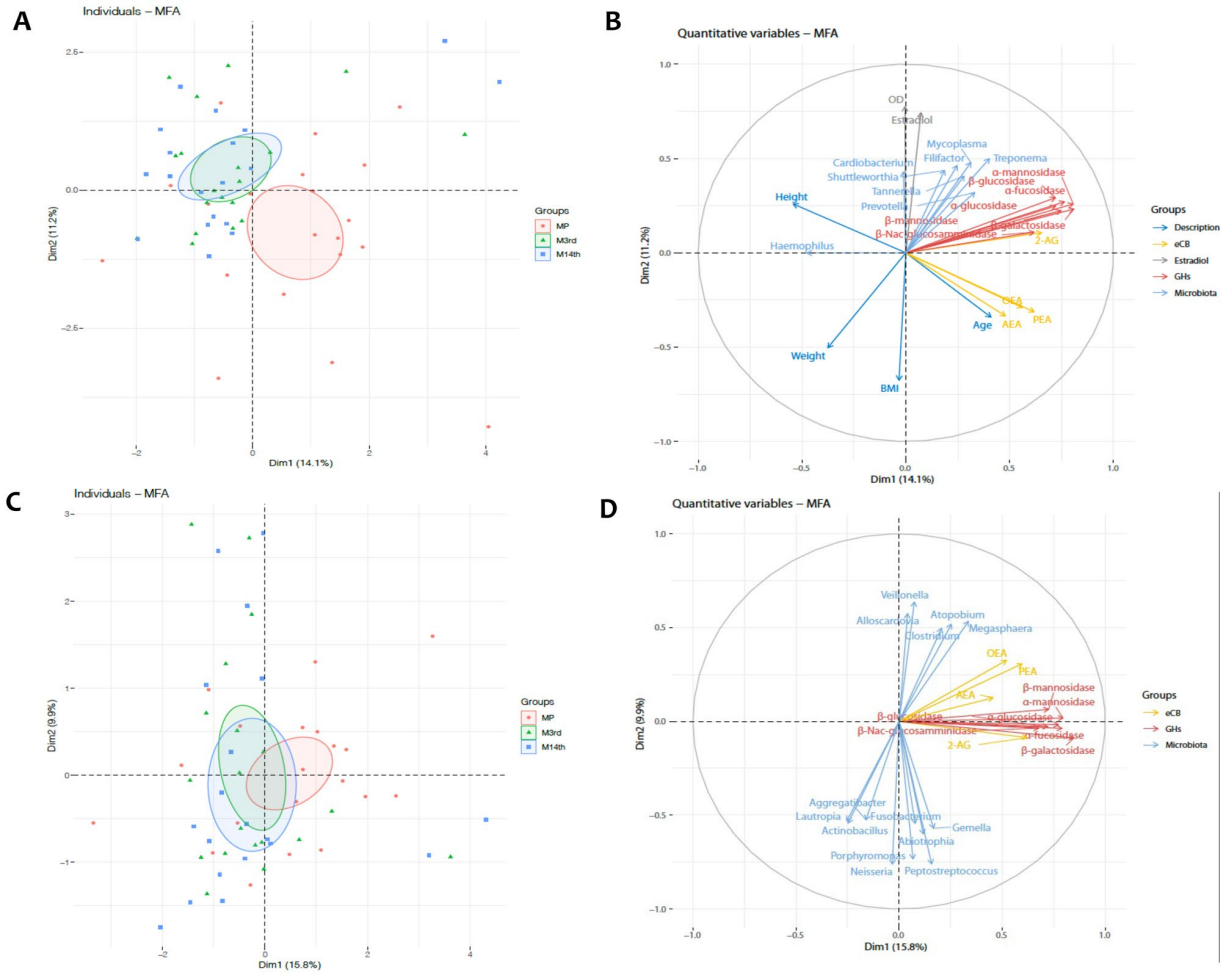
Multiple factor analysis of saliva variables. **(A)** Multiple factor analysis including variable groups meta-taxonomic analysis, glycoside hydrolases activities, NMR metabolomics and targeted lipidomics through mass spectrometry with estradiol levels, and anthropometric data show separation between experimental groups. **(B)** Importance of variables in the MFA that included all data. **(C)** Multiple factor analysis without estradiol levels and anthropometric data. **(D)** Importance of variables in the MFA that did not include estradiol levels and anthropometric data.

To better understand the variables associated with menopause, we integrated the results from meta-taxonomic analysis, glycoside hydrolases activities, NMR metabolomics and targeted lipidomics through mass spectrometry with estradiol levels, and anthropometric data using multiple factor analysis (MFA). We observed separation of the MP group from the two other groups (Fig. 8A). As expected, the main variables associated with menopause were age and anthropometrics (Fig. 8B). Aside from four microbiome genera, namely *Bulleidia, Slackia, Scardovia* and *Alloscardovia*, the omic and enzymatic approaches investigated in this study did not permit to separate the experimental groups. Indeed, when performing the analysis using only meta-taxonomic, NMR metabolomics, targeted lipidomics and glycoside hydrolases activities, it was not possible to separate the experimental groups, suggesting that these variables measured in saliva are not key players in these processes (Fig. 8C–D).

**Figure 9:**
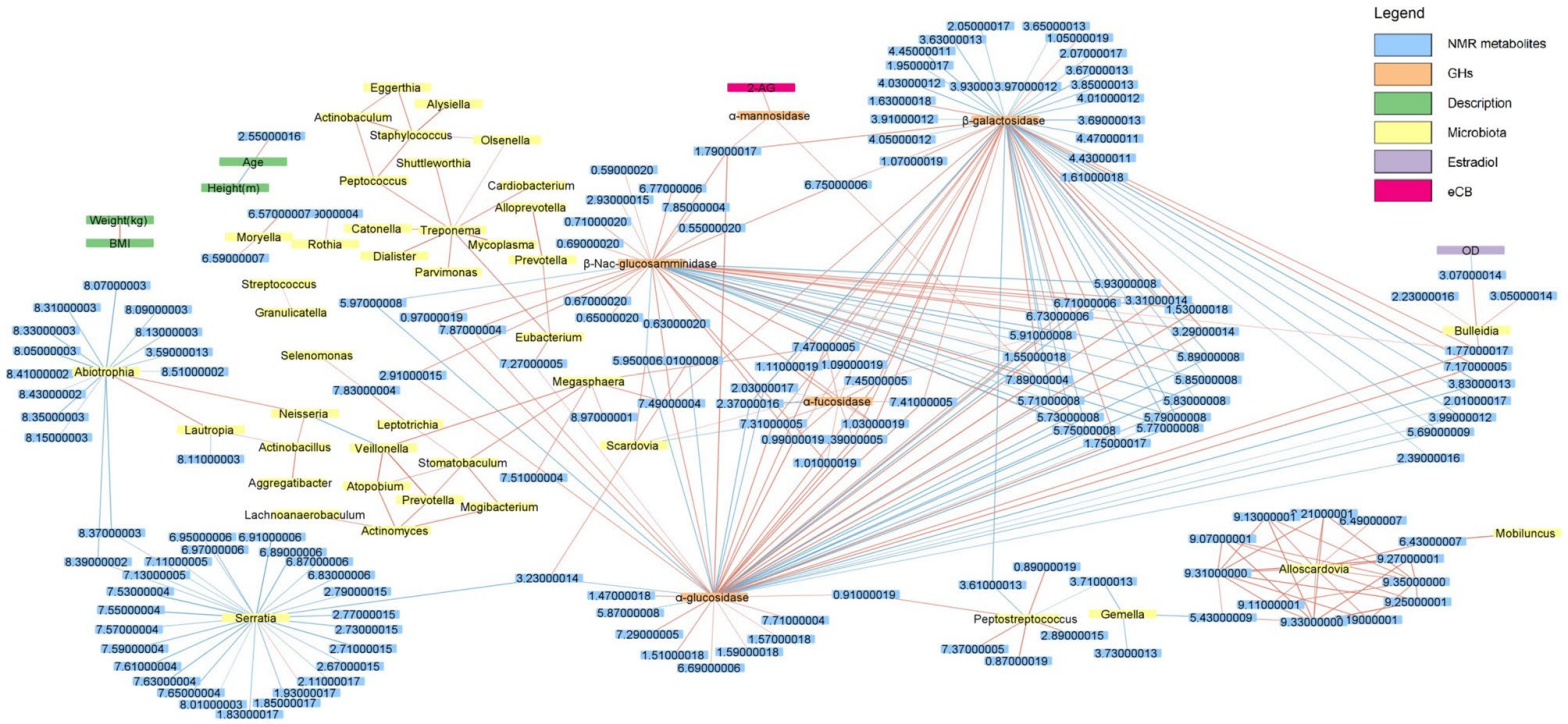
Correlation network of all variables included in the study. Spearman correlations with FDR-corrected *p* < 0.001 are shown in the network. Positive correlation is indicated by red edges and negative correlation is indicated by blue edges. Color of nodes indicate the type of variables, as shown in legend.

Nonetheless, there seemed to be potential associations between the variables we measured. We elaborated a correlation network between all the variables generated in this study (Fig. 9). This network points to many potentially central players in the saliva ecosystem. For instance, several metabolites gravitate glycoside hydrolases, including positive and negative correlations. The same is true for bacterial genera *Abiotrophica, Serratia, Peptostreptococcus* and *Alloscardovia*. We also observed two major clusters of bacteria. In addition to these general observations, the endocannabinoid 2-AG was strongly correlated with α-mannosidase. Since we observed an uneven distribution of smokers between our experimental groups, we validated if our conclusions could be affected by this lifestyle variable. Multiple factor analysis did not show differential clustering of smokers and non-smokers (Figure S8). In addition, analysis of variance do not show significant different microbiome taxa or metabolites either for smoking status or interaction between smoking and experimental groups. Therefore, we consider that smoking is not a confounding factor in this study.

## Discussion

In our study, we have observed no significant change in the oral microbiota composition between women in menopause and women with menstrual cycle. However, we report several significant findings using a multidisciplinary approach. In particular, even if no major changes in the microbiota composition of women’s saliva were observed, the genera most abundant are those ones known to be predominant in healthy individuals, such as *Streptococcus, Neisseria, Porphyromonas, Prevotella,* and *Veillonella*^25^. This is in line with the medical history of our volunteers that reported no predominant pathological issues in the general and periodontal health. Moreover, this result is comparable to a recent study in which salivary microbiome from a larger cohort of women with menstrual cycle was analysed^26^. In fact, they observed no significant differences in alpha-diversity or phase-specific clustering of the overall microbiome, even though they measure the sugar intake by weekly dietary records and it appeared to influence the composition of the salivary microbiome during the menstrual cycle^26^. Blood analysis (Fig. 1A) confirmed hormonal status typical of menopause and different stages during menstrual cycle. However, in saliva only FSH levels were detectable solely in MP group (Fig. 1B), assessing their postmenopausal status. In agreement with previous data reported in literature, the activity of β-N-Acetyl-glucosamminidase was found to be the highest in saliva samples, among the other glycoside hydrolases (GHs) screened^27^. In particular, we observed that α -glucosidase and β -N-acetyl-glucosamminidase were higher in M-14th day group as compared to M-3^r﻿﻿﻿﻿d^ day and MP samples, but no significant correlation with hormone levels were observed (Figure S3). A possible explanation of this data is the correlation between hormone levels and salivary flow, that in our project has not been investigated. In fact, recently, it was suggested a correlation between the changes in oral health as well as systemic health due to menopause and hormonal changes^28^. In particular, E2 level positively correlated with unstimulated salivary flow indicating that a decrease of E2 may result in decrease of salivary flow, which could in turn cause various oral problems associated with menopause and a possible alteration of oral microbioma. Moreover, β-N-Acetyl-glucosamminidase and α-glucosidase activities have been reported as the predominant GH not only in secreted saliva but also in healthy human dental plaque collected overnight^29^. Previous studies showed that *Prevotella intermedia, Treponema denticola, Porphyromonas gingivalis,* and *Streptococcus gordonii,* as constituent of oral microbiome, have genes coding for the β-N-acetyl-hexosaminidase and expressed this activity, which in fact indicated the presence into the oral microbiome of these organisms. In fact, the dental plaque environment favors organisms that are able to metabolize complex glycoprotein and mucins and the detection of some GH activities in human saliva have been reported and have been considered as indicator of several parameters correlated to human health^30–32^. In addition, a very recent study has also analyzed different physicochemical parameters of saliva in menopausal women, in which they did not report GH activities, but they stated that differences in saliva properties observed in menopause can potentially affect the oral environment, possibly increasing the risk of some pathological changes in the oral cavity and consequently indicating the need to take special care of this group of female patients in order to help them maintain proper oral health^33^.

Untargeted metabolomics analysis revealed a class differentiation between the menopausal group and menstrual cycle (with no difference between 3rd and 14th day). Interestingly, among metabolites that contributed to the discrimination, we found the citrate pathway and taste transduction module in which citrate plays a key role^34^. In particular, sour taste is mainly correlated with citric, malic and other acids (acetic, adipic, fumaric, lactic, succinic and tartaric) as well as the production of citrate ions^34^. The increased citrate levels in saliva of menopausal women could be responsible for the enhanced perception of sour taste. However, Saluja and co-workers have reported that both pregnant and postmenopausal women appeared to have a reduced perception of sucrose but no significant difference in the taste perception of citric acid^35^. Moreover, we also found the involvement of the galactose metabolism, where sugars contribute in taste functions, being stimulators for sweetness^36^. Fucose and galactose showed lower concentration in menopause women’s saliva which can further contribute with alteration in taste. Indeed, gustatory function and taste perception may alter eating habits and in turn modify oral microbiome affecting oral health. In addition, a galactose consumption together with low activity of galactose-1-phosphate uridyl transferase have been related to ovarian senescence^37^ and dysfunction^38^ among women non suffering with galactosemia, which is a known condition for premature ovarian infertility (POI)^39,40^. Another interesting pathway involved is the one responsible for polyamines, such as putrescine and spermidine, that are known to be produced by the intestinal microbiota and regulate multiple biological processes^41^. Interestingly, putrescine deficiency has been reported as one of the causes of poor egg quality in an aged mouse model^42^. Putrescine is produced in peri-ovulatory ovaries and its supplementation reduced egg aneuploidy, improved embryo quality, and reduced miscarriage rates in aged mice^42^. Moreover, we have observed low butyrate levels in MP group (Fig. 5F) that might be in line with the estrogen deficiency and related to specific microbial species (*Firmicutes*).

In addition, we applied a targeted lipidomics approach on saliva samples in order to investigate the possible alteration of endocannabinoid tone in association with menstrual cycle and/or menopause and oral microbiome composition. In fact, in the last decade increasing evidence have reported the cross-talk between the eCB system and the associated endocannabinoidome (eCBome), an expanded pleiotropic system that play a key role in several physio-pathological conditions, with the gut microbiome, mainly focused on metabolic and obesity-related disorders suggesting that modulation of the eCBome is related with changes in the gut bacterial community and, on the other hand, the modification of the gut microbiota by using probiotics, antibiotics or germ-free mice affected eCB signaling^20,43^. Even though the presence of the eCBs in saliva have been already reported and correlated with obesity and feeding status^44^, the potential interplay with oral microbiome and menstrual cycle and/or menopause has never been investigated. Interestingly, our data showed significant increase in 2-AG levels (Fig. 7B) in saliva of menopausal women, and no significant changes in the *N*-acylethanolamines measured. This result is in agreement with previous studies in which it is shown that plasma 2-AG is associated with menopause^45^. On the other hand, another study found an association between the Fatty Acid Amide Hydrolase (FAAH) expression in adipose tissue and anandamide circulating levels in postmenopausal women in association with obesity^46^. However, the body mass index (BMI) mean in MP group in our study does not indicate an obesity status and this may reflect in no change in AEA and the other two *N*-acylethanolamines analyzed. Recent studies further implicate the role of gut microbiota in eCBome signaling, as commensal microbe *Bacteroides* produces eCB-like *N-*acyl amides^47,48^ that are able to bind receptors activated in the eCBome^47^.

Multiple factor analysis integrating microbiome, lipidomics, glycoside hydrolases activities and metabolomics data from saliva showed some, but very little, difference between the three groups. These results suggest that saliva may not harbor key biomarkers associated with menopause.

Some studies reviewed by Segovia-Mendoza and Morales-Montor reported a direct correlation between estradiol and the release of β-hexosaminidase in mast cell line^49^. Moreover, children with type 1 diabetes showed an increased salivary β-hexosaminidase concentration which may be useful in the diagnosis^50^. In addition, β-D-hexosaminidase seems to play a key role in the biofilm formation in saliva of some oral pathogen^51^. It is possible that the β-hexosaminidase activity has effects not only on interactions with saliva-coated surfaces, but also interactions with host epithelial cells. These enzymes may also influence interactions with other bacteria in terms of release of glucose or galactose that may act as growth substrates for cohabiting bacteria.

Our data shows that although no significant alterations in the composition of the salivary microbiome was detected, potential associations between the variables measured exist, such as the enzymatic activity of some glycoside hydrolases and the presence of specific metabolites. Multi-omics analysis did not show any significant discrimination combining simultaneously all experimental data, but revealed some metabolites and enzymes as the most responsible for the distribution.

In conclusion, the use of a multidisciplinary approach allowed an exhaustive investigation of the oral microbiome of healthy women and provided some indication about microbiome-derived predictive biomarkers that will potentially help in finding novel strategies to establish the correct hormonal balance in post-menopausal women.

## Methods

### Volunteers

The study was conducted as per the guidelines of Helsinki declaration and received the ethical clearance from CNR Ethical Commission (Prot. n. 3419). All participants provided written informed consent before enrolment in the study sample. Thirty-nine medically healthy women aged 26–77 years (19 with menstrual cycle and 20 in menopause) were recruited. Participants completed questionnaires assessing detailed medical and medication history and demographic characteristics (Table 1). No financial incentive was offered to any of the survey participants.

**Table 1:** Demographic, lifestyle, and menstrual cycle characteristics of women enrolled in the AMICA study.

Subject	Demographics		Menstrual cycle characteristics			Lifestyle habits		
	Age	BMI	Lenght (days)	Duration (days)	Oral contraceptives or HRT	Physical activity^a^	Current smoker	Mediterranean diet
M1	32	22,7	30	6	no	Low	no	yes
M2	41	21,5	28	5	no	Low	yes	yes
M3	39	18,0	28	6	no	Low	yes	yes
M5	50	22,5	28	5	no	Low	yes	yes
M6	41	20,3	28	5	no	Moderate	no	yes
M8	46	20,1	30	4	no	Moderate	no	yes
M9	28	26,0	30	3	no	Low	no	yes
M10	38	20,9	27	4	no	Low	yes	yes
M11	29	22,1	28	5	no	Low	yes	yes
M12	35	25,0	28	5	no	Low	yes	yes
M13	50	25,0	30	6	no	Low	no	yes
M14	28	20,8	28	4	no	Low	no	yes
M15	32	31,2	30	3	no	Low	no	yes
M16	39	21,6	30	4	no	Low	no	yes
M17	28	23,5	29	4	no	Low	no	yes
M18	26	22,3	30	5	no	Moderate	yes	yes
M19	40	20,0	28	5	no	Moderate	yes	yes
M20	35	22,4	28	4	no	Moderate	yes	yes
M21	46	26,0	30	4	no	Moderate	no	yes
MP1	59	21,8	–	–	no	Low	no	yes
MP2	71	24,0	–	–	no	Low	no	yes
MP3	67	28,7	–	–	no	Low	no	yes
MP4	57	25,4	–	–	no	Low	no	yes
MP5	52	24,4	–	–	no	Low	no	yes
MP6	62	24,7	–	–	no	Low	no	yes
MP7	74	27,3	–	–	no	Low	no	yes
MP8	70	27,3	–	–	no	Low	no	yes
MP9	77	27,6	–	–	no	Low	no	yes
MP10	52	25,7	–	–	no	Low	yes	yes
MP11	66	21,5	–	–	no	Low	yes	yes
MP12	67	23,9	–	–	no	Low	no	yes
MP13	56	22,0	–	–	no	Low	no	yes
MP14	55	26,4	–	–	no	Low	no	yes
MP15	54	27,7	–	–	no	Low	no	yes
MP16	66	32,0	–	–	no	Low	no	yes
MP17	51	21,9	–	–	no	Low	yes	yes
MP18	52	26,3	–	–	no	Low	no	yes
MP19	66	18,5	–	–	no	Low	no	yes
MP20	68	26,0	–	–	no	Low	no	yes

*M* menstrual cycle; *MP* menopause; *BMI* body mass index; *HRT* hormone replacement therapy.

^a^Low: 30 min per week; Moderate: one or two hours per week.

In brief a total of 39 healthy women were enrolled in the study: 19 with menstrual cycle and 20 post-menopausal women.

*Inclusion criteria*

- Females (18–80 years),
- To be able to understand and communicate in Italian,
- To be able to give informed consent

*Exclusion criteria*

- Patients who underwent to dental care within the last 30 days
- Patients who showed an irregular menstrual cycle or a cycle with a length more than 30 days,
- Current infective disease or fever, nausea, diarrhea within the last 30 days,
- Current treatment with hormonal replacement therapy,
- Use of antibiotics within the last 30 days,
- Patients who are pregnant or lactating or aborted within the last 6 months,
- Patients who are using hormonal contraceptives

The volunteers underwent blood sampling for the determination of female sex hormones [follicle stimulating hormone (FSH), estradiol (E2), luteinizing hormone (LH), progesterone (PGN), Biochemios Lab, Pozzuoli (NA)] and saliva sampling for the analysis of the oral microbiome. Saliva samples in women of reproductive age were collected during ovulation (M-14th and M-15th) and during the menstrual cycle (M-3rd), using the protocol below.

### Samples collection

Saliva samples were collected unstimulated in a sterile tube after an overnight fast without tooth brushing. After collection, the salivary samples were immediately frozen on dry ice and stored at − 80 °C until analysis.

### Salivary hormone levels

The levels of female sex hormones (FSH, E2, LH and PGN ) were measured in the saliva samples, in the different experimental conditions, by SinglePlex ELISA kit with a GENios-Pro Reader (Tecan) following the manufacturer's instructions.

### Next generation sequencing for salivary microbiota composition

Bacterial DNA was purified from salivary samples with the use of “QIAamp® DNA Microbiome Kit” which allows depletion of human host DNA from biological samples and yields enriched bacterial DNA. Purification protocol consists of a degradation of host DNA followed by a disruption of bacterial cells through a combination of mechanical and chemical lysis. Target DNA is then purified through adsorption to a silica membrane, washing steps and elution with appropriate buffer. Purified DNA was accurately quantified using a fluorescence-based quantification method, such as Qubit dsDNA HS and 5 ng DNA for each sample were used for amplification. Library production was carried out by the Ion 16S™ Metagenomics Kit, this kit contains primer pools for PCR amplification of 7 hypervariable regions of the 16S rDNA gene of bacteria. Amplicon libraries were then sequenced with the Ion PGM™ sequencing 400 kit on the Ion PGM™ platform and analyzed using the Ion 16S™ metagenomics analyses module within the Ion Reporter™ software. The analysis software used 2 different databases: Curated Greengenes and Premium Curated MicroSEQ ID 16 s rRNA. Raw sequencing reads are available in SRA (PRJNA897843).

In addition, OPLS-DA approach was also applied to taxa data matrix with observations in row and microbiota families UV (unit variance) normalized in columns. Such analysis was carried out to explore possible presence of dominant families according to subject’s specific menstrual cycle time point (3rd day, 14th day of fertile women’s menstrual cycle, menopause women).

### NMR-based metabolomics

Saliva samples were thawed, centrifugated and 400 µL of salivary supernatant were mixed with 300 µL of Phosphate Buffer Saline (PBS, pH 7.4) with 10% D2O for lock procedure, and then transferred into an NMR tube. Final pH is on average 7.2–7.6. One-dimensional (1D-1H) spectra were recorded at 600.13 MHz on a Bruker Avance III-600 spectrometer equipped with a TCI CryoProbeTM fitted with a gradient along the Z-axis, at a probe temperature of 27 °C, using the excitation sculpting sequence for solvent H_2_O suppression ^52^. Spectra were referred to internal 0.1 mM sodium trimethylsilylpropionate (TSP), assumed to resonate at δ = 0.00 ppm. For each sample, the metabolic profile was attained through the acquisition of proton spectra (1D-NMR) and 2D NMR mono and hetero nuclear experiments (TOCSY, HSQC), useful for the recognition and assignment of metabolites. Subsequently, all the one-dimensional spectra undergone to multivariate statistical data analysis with unsupervised and supervised regression methods in order to discriminate the classes of samples (fertile subjects / subjects in menopause) on the basis of the changes in their corresponding metabolic profile induced by the different hormone levels.

### Multivariate data analysis

Saliva proton spectra ranging from 9.50 ppm to 0.50 ppm were automatically binned into 450 integrals of 0.02 ppm each using the AMIX 3.9.15 software package (Bruker Biospin GmbH, Rheinstetten, Germany). The residual water resonance region (5.05–4.55 ppm) was excluded, and each integrated region was normalized to the total spectrum area to avoid possible dilution effects on the signals. The obtained NMR data format, expressed by a matrix (X matrix), was firstly imported into mixOmics routine in R^19^, after UV scaling, to analyze with a multilevel PLS-DA approach the salivary metabolic variations of the reduced dataset only consisting of fertile subjects at the 3rd and 14th day of their menstrual cycle. Then, the whole X matrix was imported into SIMCA-P + 14 package (Umetrics, Umeå, Sweden) where Principal Components Analysis (PCA) and Orthogonal Projections to Latent Structures Discriminant Analysis (OPLS-DA) were performed, after UV scaling. We first applied PCA-class to explore data trend and eventually exclude outliers (data not shown). Once homogeneity was assessed, we applied supervised OPLS-DA to investigate the metabolic differences in saliva collected in three diverse feminine hormonal conditions. Initially, PCA and PCA-class was used to reduce data dimensionality and to explore possible trends as well as the existence of outliers. Once class homogeneity was assessed for each group, supervised OPLS-DA was applied to emphasize categories discrimination, where dummy variables were assigned to define class belonging (Y matrix). Supervised regressions were conducted comparing saliva of fertile groups at different time of their cycle (3rd and 14th day) and menopausal class, in order to generate predictive models that better relate metabolites variation to the different hormonal assessments. Each model quality was evaluated by using the goodness-of-fit parameter (R2) and the goodness-of-prediction parameter (Q2)^53^ together with an internal iterative 7-round cross-validation and permutation test (800 repeats). The discriminatory metabolites were selected based on the bins containing NMR signals which were not overlapping with other peaks for the relative quantification, performed using OriginPro 9.1 software package (OriginLab Corporation, Northampton, USA). Statistical significance for selected metabolites was determined by parametric (ANOVA with Tukey correction) or non-parametric (Mann–Whitney U) tests according to the results of normality test performed on data to evaluate each distribution (Shapiro–Wilk, Kolgomorov-Smirnov test). P values < 0.05 were considered as statistically significant. Moreover, spectroscopic data were integrated with the enzyme expressions evaluated for the glycosidase activity. For this purpose, a correlation map with hierarchical clustering was also generated by combining clinical test values and selected bin integrals of significant metabolites with R software^54^. The Euclidean distance was considered for the metrics and the centroid method for clustering criterion.

### Network analysis

Enrichment analysis on selected and more representative metabolites found in fertile/menopause class separation was applied using the ‘pagerank’ method computed by the FELLA package in R^55,56^. Starting from the set of altered compounds, such analysis suggests affected reactions, enzymes, modules and pathways using label propagation in a knowledge model network based on Homo sapiens database in KEGG^57–59^. The resulting network and subnetwork are visualized and exported in related plot and table with a threshold of *p* < 0.001.

### Targeted lipidomics

Saliva samples collected according to the protocol reported above were subjected to a series of biochemical steps for the extraction, purification and quantification of endocannabinoids, as reported in the literature ^18^. The extracts or fractions enriched in endocannabinoids were then analyzed by liquid chromatography coupled with mass spectrometry by chemical ionization at atmospheric pressure (LC-APCI-MS) as reported above^19^

### Screening of glycosyde hydrolases activities in human female saliva

Samples were thawed, centrifuged and the supernatants were diluted tenfold with Tris buffer 100 mM, pH 7,5^27^. These salivary supernatants were analyzed for the presence of glycosidase activities:

- α-mannosidase
- β-mannosidase
- α -fucosidase
- b-galactosidase
- α -glucosidase
- β -Nac-glucosamminidase
- β -glucosidase.

GH activities were measured separately for each saliva sample using black 96-well microtiter plates (BD Bioscience). Aliquots (50 µL) of diluted saliva samples were added to 20 µL of 4-nitrophenyl (PNP) substrates solution in Tris buffer 100 mM, pH 7,5 to a final concentration of 4 µM. Plates were incubated at 37 °C for 1 h, before the reaction was quenched with 130 µL of 500 mM Na_2_CO_3_-NaHCO_3_ buffer (pH 10.3).

### Statistical analysis

Data were represented as mean ± SEM. Plasmatic hormones and glycoside hydrolases were analyzed using the Two-way ANOVA followed by Tukey post hoc test. eCB and related *N*-acylethanolamine levels were analyzed using one-way ANOVA, followed by Tukey post hoc comparisons.

Statistical analysis of 16S microbiome profiling was performed at the rank of genera using linear model followed by one-way ANOVA on rank-transformed relative abundances. Analyses were performed with and without 20,000 reads rarefaction with similar results. Taxa were considered significantly modulated if FDR-corrected p-values were lower than 0.05.

### Multi-omics analysis

Integrative analysis of meta-taxonomic analysis, glycoside hydrolases activities, NMR metabolomics and targeted lipidomics through mass spectrometry with E2 levels, and anthropometric data was performed using multiple factor analysis (MFA) from the FactoMiner package^60^. Variables were scaled to unit variance for MFA analysis. Prior to the construction of the correlation network, we scaled, centered and applied Yeo-Johnson transformation to reduce biases in the correlations. Spearman correlations were performed in Rstudio 1.3.1093. *P*-values were FDR corrected and only correlations with FDR-corrected *p* < 0.001 were included in the network. The network was build using Cytoscape 3.8.0. Node position was manually adjusted to improve visualization.

### Ethics approval and consent to participate

The study was conducted as per the guidelines of Helsinki declaration and received the ethical clearance from CNR Ethical Commission (Prot. n. 3419).

## Supplementary Information

Supplementary Information 1.

Supplementary Information 2.

Supplementary Information 3.

Supplementary Information 4.

Supplementary Information 5.

Supplementary Information 6.

Supplementary Information 7.

Supplementary Information 8.

Supplementary Information 9.

Supplementary Information 10.

Supplementary Information 11.

Supplementary Information 12.

Supplementary Information 13.

Supplementary Information 14.

## Data Availability

All data generated or analysed during this study are included in this published article and its supplementary information files.
