# Supplementary figures and images for: Analysis of the oral microbiome during hormonal cycle and its alterations in menopausal women: the “AMICA” project
Source: Sci Rep. 2022 Dec 21;12:22086. doi: 10.1038/s41598-022-26528-w (PMC9772230; doi:10.1038/s41598-022-26528-w)

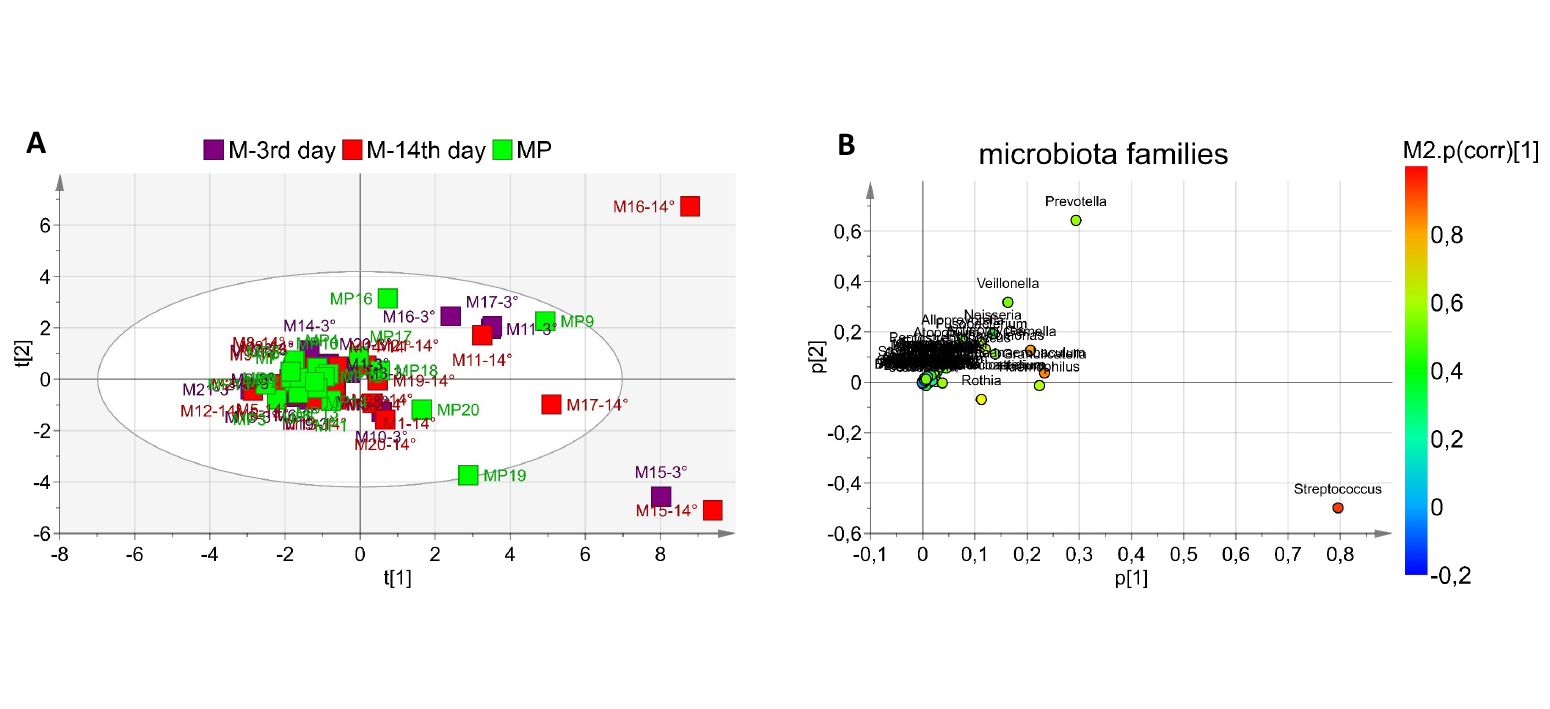

Supplement: Supplementary file 1 — Supplementary Information 1. [file 41598_2022_26528_MOESM1_ESM.tif]

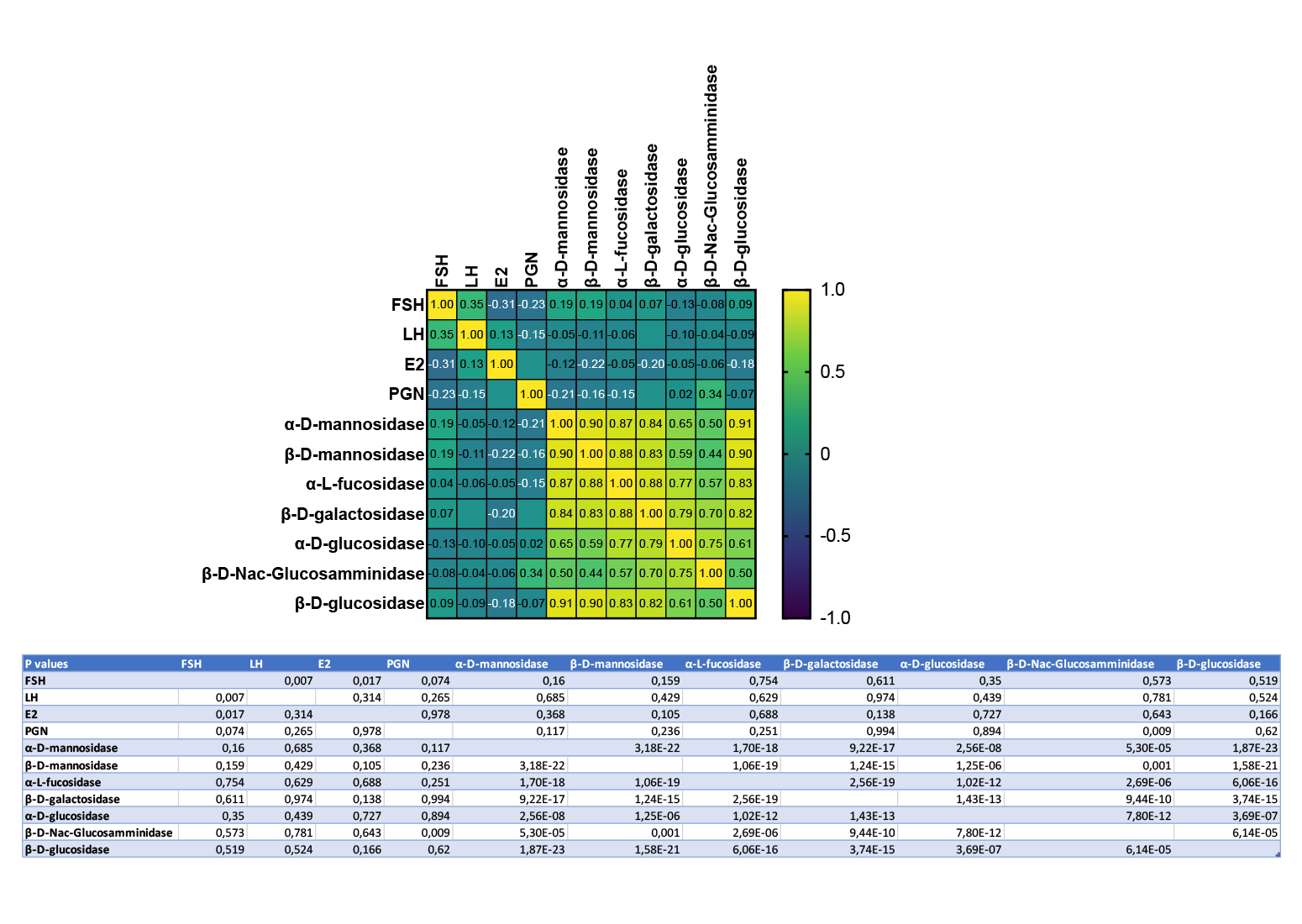

Supplement: Supplementary file 3 — Supplementary Information 3. [file 41598_2022_26528_MOESM3_ESM.tif]

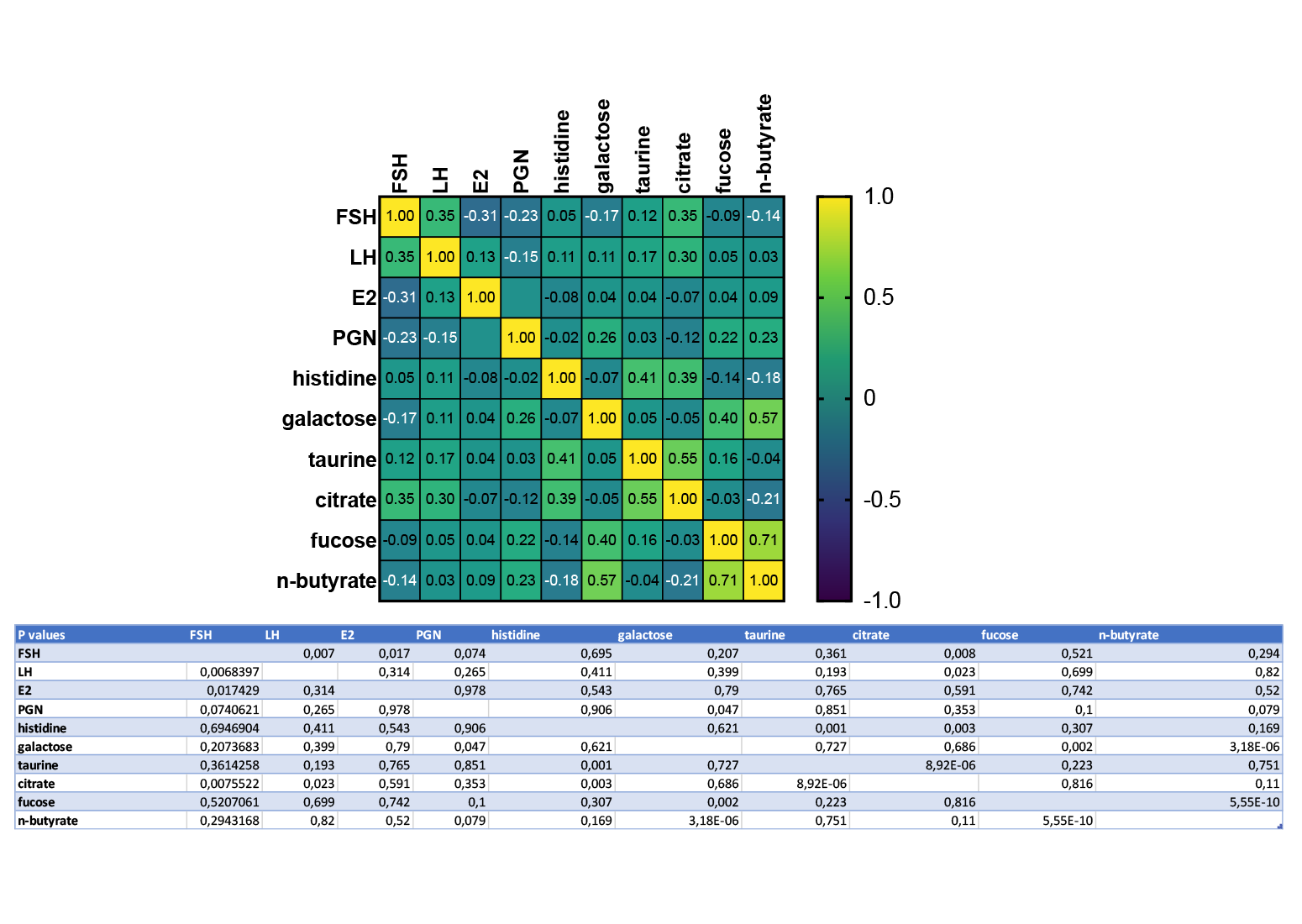

Supplement: Supplementary file 4 — Supplementary Information 4. [file 41598_2022_26528_MOESM4_ESM.tif]

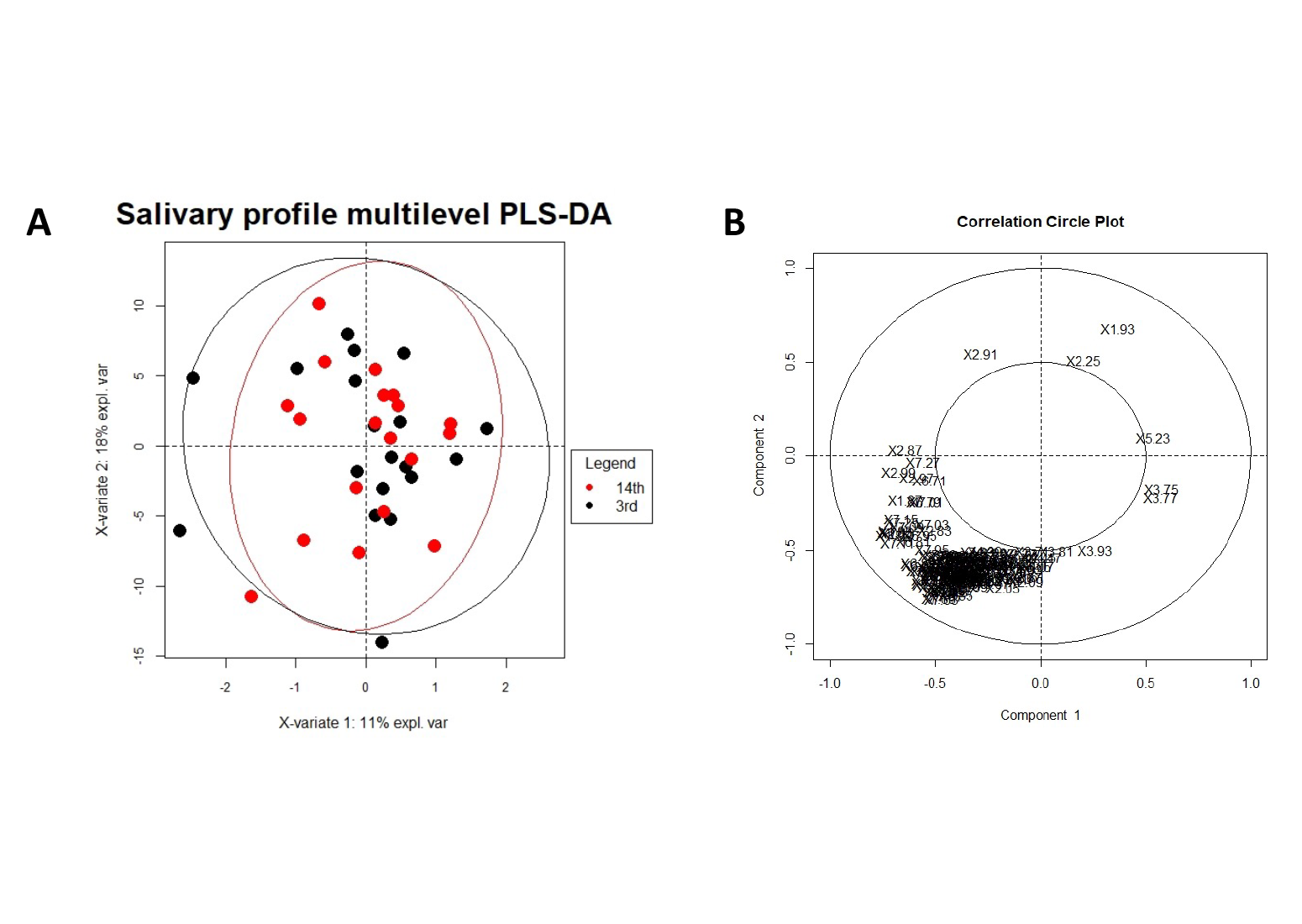

Supplement: Supplementary file 5 — Supplementary Information 5. [file 41598_2022_26528_MOESM5_ESM.tif]

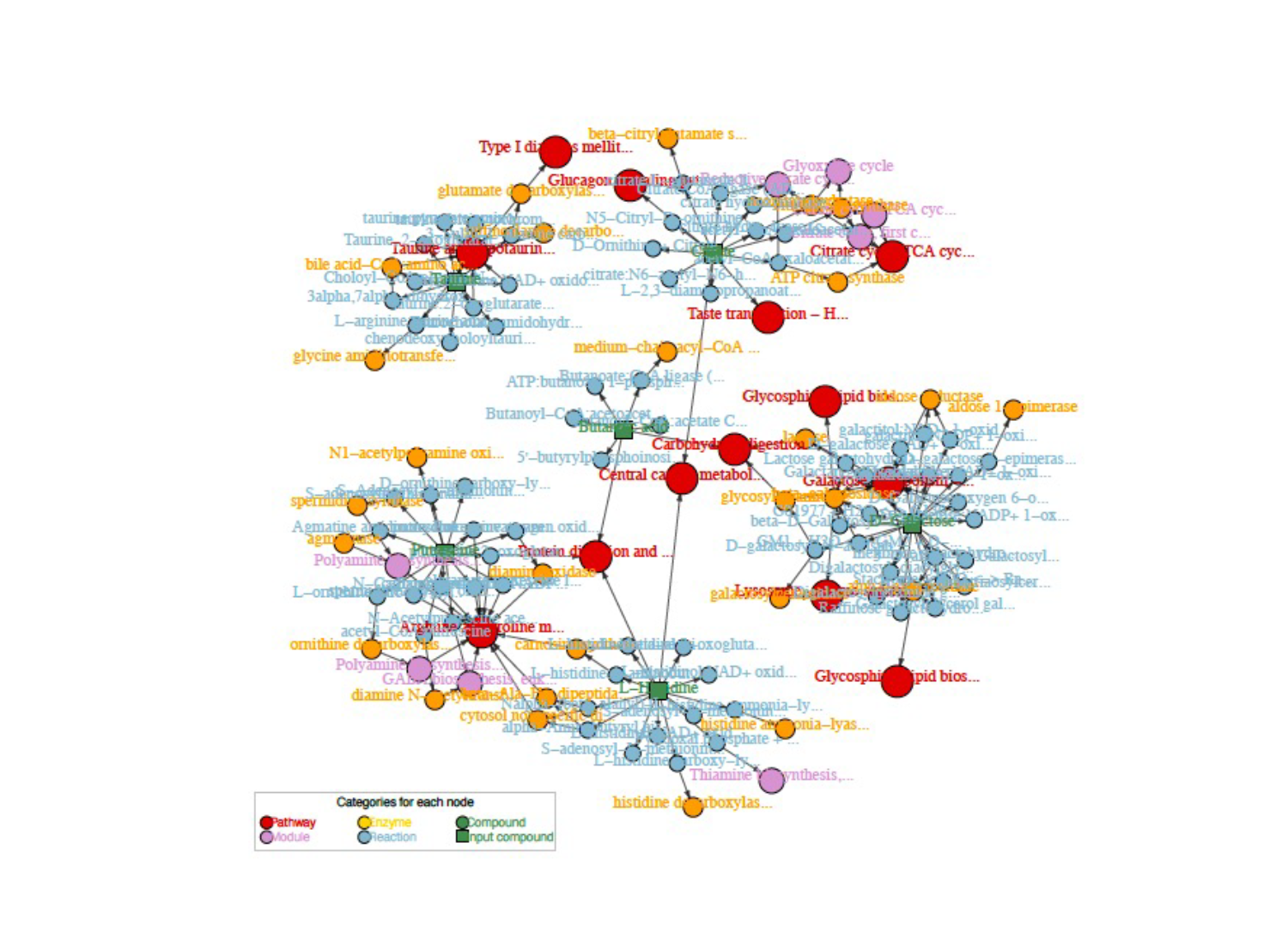

Supplement: Supplementary file 6 — Supplementary Information 6. [file 41598_2022_26528_MOESM6_ESM.tif]

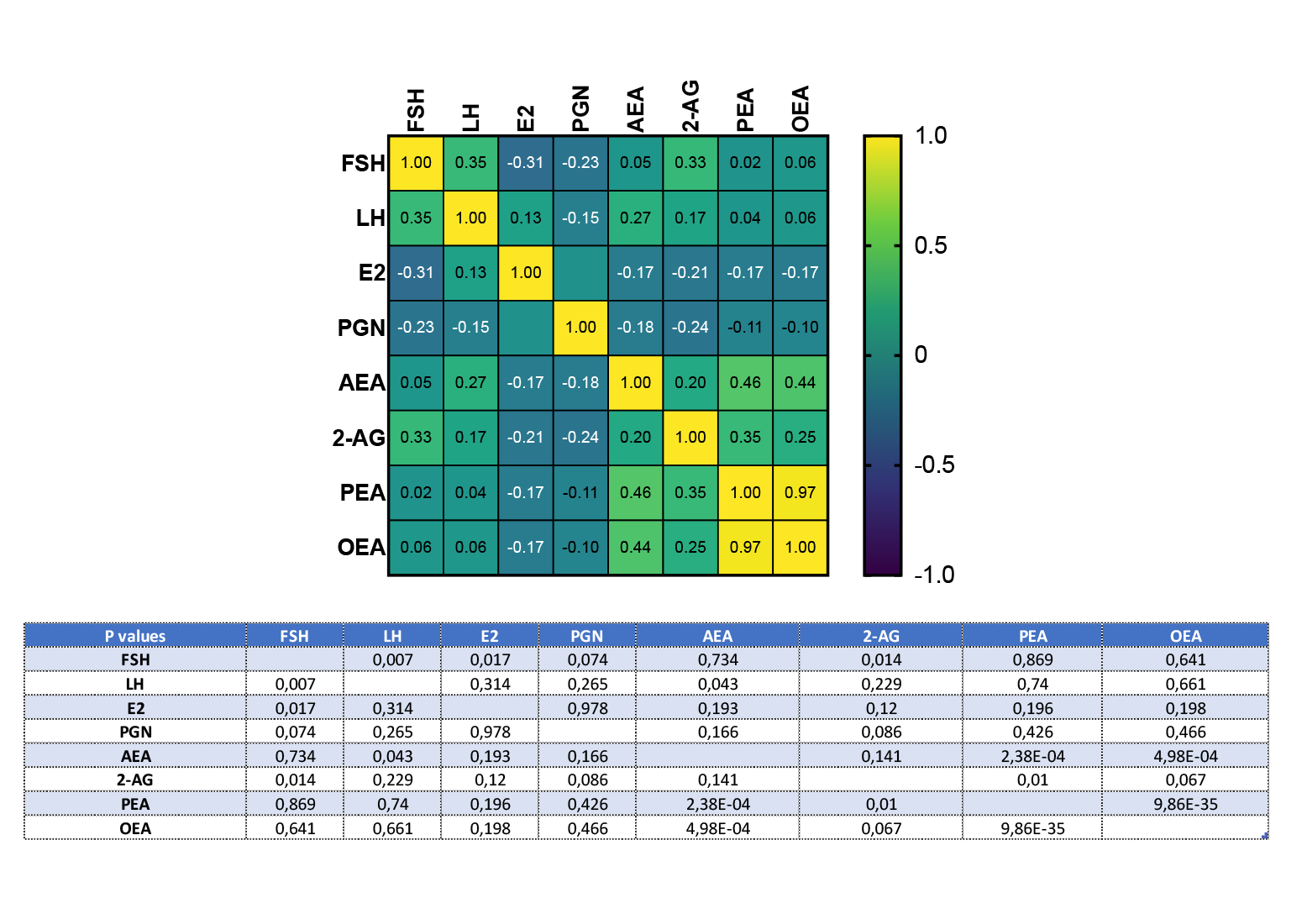

Supplement: Supplementary file 7 — Supplementary Information 7. [file 41598_2022_26528_MOESM7_ESM.tif]
