# Supplementary material for: Analysis of the oral microbiome during hormonal cycle and its alterations in menopausal women: the “AMICA” project
Source: Sci Rep. 2022 Dec 21;12:22086. doi: 10.1038/s41598-022-26528-w (PMC9772230; doi:10.1038/s41598-022-26528-w)
Supplement: Supplementary file 9 — Supplementary Information 9. [file 41598_2022_26528_MOESM9_ESM.docx]

**Supplementary Table S1.**

| Hormones | M3^rd^ day | | M14^th^ day | | MP | |
| --- | --- | --- | --- | --- | --- | --- |
|  | mean | SEM | mean | SEM | mean | SEM |
| E2 | 1,7315 | 0,0009 | 1,7315 | 0,0009 | 1,7295 | 0,0015 |
| PGN | 2,1652 | 0,0001 | 2,1655 | 0,00005 | 2,1655 | 3,149E-05 |
| LH | 0,04 | 0,02 | 0,05 | 0,03 | 0,06 | 0,01 |
