# Supplementary material for: Analysis of the oral microbiome during hormonal cycle and its alterations in menopausal women: the “AMICA” project
Source: Sci Rep. 2022 Dec 21;12:22086. doi: 10.1038/s41598-022-26528-w (PMC9772230; doi:10.1038/s41598-022-26528-w)
Supplement: Supplementary file 10 — Supplementary Information 10. [file 41598_2022_26528_MOESM10_ESM.docx]

**Supplementary Table S2.**

| Families | M3^rd^ day | | M14^th^ day | | MP | |
| --- | --- | --- | --- | --- | --- | --- |
|  | **mean** | **sem** | **mean** | **sem** | **mean** | **sem** |
| Streptococcaceae | 39,029 | 2,990 | 40,504 | 3,024 | 38,287 | 3,225 |
| Neisseriaceae | 7,554 | 1,966 | 7,895 | 1,639 | 8,963 | 1,728 |
| Prevotellaceae | 16,444 | 1,955 | 14,132 | 1,782 | 16,479 | 1,469 |
| Veillonellaceae | 7,359 | 1,011 | 6,944 | 1,097 | 6,326 | 0,891 |
| Bacillales incertae sedis | 2,997 | 0,407 | 3,211 | 0,442 | 3,572 | 0,428 |
| Carnobacteriaceae | 3,566 | 0,279 | 3,936 | 0,469 | 4,328 | 0,431 |
| Aerococcaceae | 0,305 | 0,194 | 0,276 | 0,088 | 0,178 | 0,059 |
| Porphyromonadaceae | 3,671 | 0,722 | 3,188 | 0,623 | 2,871 | 0,533 |
| Pasteurellaceae | 8,114 | 1,140 | 9,388 | 1,110 | 8,185 | 1,727 |
| Fusobacteriaceae | 2,059 | 0,307 | 1,693 | 0,291 | 1,428 | 0,180 |
| Actinomycetaceae | 1,435 | 0,242 | 1,695 | 0,297 | 2,061 | 0,340 |
| Peptostreptococcaceae | 0,623 | 0,137 | 0,534 | 0,120 | 0,463 | 0,078 |
| Flavobacteriaceae | 0,377 | 0,118 | 0,433 | 0,148 | 0,263 | 0,047 |
| Clostridiales Family XI. Incertae Sedis | 0,339 | 0,072 | 0,297 | 0,055 | 0,528 | 0,092 |
| Lachnospiraceae | 0,644 | 0,095 | 0,578 | 0,112 | 0,724 | 0,152 |
| Eubacteriaceae | 0,165 | 0,062 | 0,103 | 0,036 | 0,084 | 0,037 |
| Lactobacillaceae | 0,535 | 0,049 | 0,567 | 0,072 | 0,652 | 0,070 |
| Clostridiaceae | 0,726 | 0,104 | 0,581 | 0,119 | 0,623 | 0,110 |
| Corynebacteriaceae | 0,082 | 0,025 | 0,099 | 0,044 | 0,251 | 0,074 |
| Coriobacteriaceae | 0,553 | 0,131 | 0,574 | 0,156 | 0,561 | 0,136 |
| Clostridiales Family XIII. Incertae Sedis | 0,435 | 0,084 | 0,405 | 0,087 | 0,537 | 0,092 |
| Micrococcaceae | 0,893 | 0,159 | 1,084 | 0,204 | 1,018 | 0,143 |
| Spirochaetaceae | 0,096 | 0,052 | 0,082 | 0,043 | 0,049 | 0,020 |
| Erysipelotrichaceae | 0,167 | 0,025 | 0,135 | 0,021 | 0,256 | 0,053 |
| Burkholderiaceae | 0,036 | 0,016 | 0,053 | 0,020 | 0,085 | 0,025 |
| Leptotrichiaceae | 0,610 | 0,144 | 0,575 | 0,133 | 0,475 | 0,108 |
| Sphingobacteriaceae | 0,016 | 0,009 | 0,007 | 0,004 | 0,000 | 0,000 |
| Mycoplasmataceae | 0,030 | 0,015 | 0,022 | 0,012 | 0,012 | 0,008 |
| Campylobacteraceae | 0,388 | 0,075 | 0,261 | 0,061 | 0,220 | 0,032 |
| Staphylococcaceae | 0,007 | 0,007 | 0,006 | 0,006 | 0,000 | 0,000 |
| [Paraprevotellaceae] | 0,082 | 0,038 | 0,081 | 0,029 | 0,112 | 0,062 |
| Bacillaceae | 0,014 | 0,007 | 0,003 | 0,002 | 0,011 | 0,004 |
| Cardiobacteriaceae | 0,015 | 0,006 | 0,012 | 0,006 | 0,004 | 0,002 |
| Bifidobacteriaceae | 0,058 | 0,033 | 0,108 | 0,089 | 0,015 | 0,013 |
| Bdellovibrionaceae | 0,015 | 0,006 | 0,007 | 0,003 | 0,003 | 0,002 |
| Thermogemmatisporaceae | 0,008 | 0,004 | 0,005 | 0,003 | 0,002 | 0,001 |
| Ruminococcaceae | 0,009 | 0,004 | 0,007 | 0,003 | 0,012 | 0,004 |
| Dehalococcoidaceae | 0,012 | 0,005 | 0,006 | 0,002 | 0,005 | 0,004 |
| Bacteroidaceae | 0,009 | 0,004 | 0,008 | 0,004 | 0,010 | 0,008 |
| Propionibacteriaceae | 0,006 | 0,003 | 0,004 | 0,002 | 0,007 | 0,004 |
| Peptoniphilaceae | 0,147 | 0,041 | 0,112 | 0,032 | 0,103 | 0,030 |
| Christensenellaceae | 0,003 | 0,002 | 0,000 | 0,000 | 0,002 | 0,002 |
| Peptococcaceae | 0,001 | 0,001 | 0,003 | 0,001 | 0,001 | 0,001 |
| Helicobacteraceae | 0,006 | 0,003 | 0,004 | 0,002 | 0,004 | 0,002 |
| Clostridiales Family XII. Incertae Sedis | 0,002 | 0,001 | 0,003 | 0,002 | 0,001 | 0,001 |
| Sphingomonadaceae | 0,069 | 0,017 | 0,054 | 0,016 | 0,050 | 0,014 |
| Holosporaceae | 0,004 | 0,004 | 0,003 | 0,003 | 0,000 | 0,000 |
| Erythrobacteraceae | 0,019 | 0,007 | 0,019 | 0,009 | 0,007 | 0,004 |
| Rikenellaceae | 0,001 | 0,001 | 0,004 | 0,003 | 0,000 | 0,000 |
| Comamonadaceae | 0,000 | 0,000 | 0,005 | 0,004 | 0,001 | 0,001 |
| Anaplasmataceae | 0,000 | 0,000 | 0,001 | 0,001 | 0,000 | 0,000 |
| Rhodospirillaceae | 0,000 | 0,000 | 0,001 | 0,001 | 0,000 | 0,000 |
| Rhizobiaceae | 0,000 | 0,000 | 0,001 | 0,001 | 0,000 | 0,000 |
| Geobacteraceae | 7,789 | 4,016 | 9,614 | 5,352 | 9,733 | 5,754 |
| Hyphomicrobiaceae | 0,000 | 0,000 | 0,001 | 0,001 | 0,000 | 0,000 |
| Oxalobacteraceae | 0,004 | 0,002 | 0,005 | 0,002 | 0,004 | 0,003 |
| Microthrixaceae | 0,001 | 0,001 | 0,000 | 0,000 | 0,000 | 0,000 |
| Moraxellaceae | 0,041 | 0,041 | 0,007 | 0,007 | 0,000 | 0,000 |
| Flammeovirgaceae | 0,001 | 0,001 | 0,000 | 0,000 | 0,000 | 0,000 |
| Synergistaceae | 0,004 | 0,004 | 0,001 | 0,001 | 0,002 | 0,002 |
| Beijerinckiaceae | 0,001 | 0,001 | 0,000 | 0,000 | 0,000 | 0,000 |
| Xanthomonadaceae | 0,000 | 0,000 | 0,004 | 0,004 | 0,000 | 0,000 |
| Enterobacteriaceae | 0,189 | 0,053 | 0,253 | 0,057 | 0,155 | 0,046 |
| Cytophagaceae | 0,000 | 0,000 | 0,001 | 0,001 | 0,000 | 0,000 |
| Idiomarinaceae | 0,002 | 0,002 | 0,002 | 0,002 | 0,000 | 0,000 |
| Sutterellaceae | 0,002 | 0,002 | 0,000 | 0,000 | 0,001 | 0,001 |
| Rickettsiaceae | 0,004 | 0,004 | 0,003 | 0,003 | 0,000 | 0,000 |
| Ferritrophicaceae | 0,003 | 0,003 | 0,000 | 0,000 | 0,000 | 0,000 |
| Nocardioidaceae | 0,000 | 0,000 | 0,003 | 0,003 | 0,000 | 0,000 |
| Hyphomonadaceae | 0,000 | 0,000 | 0,001 | 0,001 | 0,000 | 0,000 |
| Caulobacteraceae | 0,000 | 0,000 | 0,002 | 0,002 | 0,000 | 0,000 |
| Spiroplasmataceae | 0,007 | 0,006 | 0,001 | 0,001 | 0,003 | 0,002 |
