# Supplementary material for: Analysis of the oral microbiome during hormonal cycle and its alterations in menopausal women: the “AMICA” project
Source: Sci Rep. 2022 Dec 21;12:22086. doi: 10.1038/s41598-022-26528-w (PMC9772230; doi:10.1038/s41598-022-26528-w)
Supplement: Supplementary file 11 — Supplementary Information 11. [file 41598_2022_26528_MOESM11_ESM.docx]

**Supplementary Table S3.**

| Genera | M3^rd^ day | | M14^th^ day | | MP | |
| --- | --- | --- | --- | --- | --- | --- |
|  | **mean** | **sem** | **mean** | **sem** | **mean** | **sem** |
| Streptococcus | 41,403 | 2,979 | 42,988 | 3,050 | 40,525 | 3,257 |
| Prevotella | 14,764 | 1,987 | 12,427 | 1,814 | 14,983 | 1,482 |
| Neisseria | 7,897 | 2,126 | 8,237 | 1,792 | 9,436 | 1,808 |
| Haemophilus | 7,136 | 1,020 | 8,276 | 1,008 | 7,219 | 1,508 |
| Veillonella | 7,126 | 0,963 | 6,993 | 1,069 | 6,259 | 0,874 |
| Granulicatella | 3,622 | 0,301 | 4,046 | 0,497 | 4,373 | 0,459 |
| Gemella | 3,244 | 0,448 | 3,469 | 0,479 | 3,795 | 0,443 |
| Porphyromonas | 3,284 | 0,648 | 2,767 | 0,544 | 2,565 | 0,481 |
| Fusobacterium | 2,203 | 0,338 | 1,813 | 0,314 | 1,532 | 0,208 |
| Actinomyces | 1,508 | 0,252 | 1,772 | 0,308 | 2,152 | 0,354 |
| Rothia | 0,945 | 0,166 | 1,145 | 0,212 | 1,079 | 0,149 |
| Abiotrophia | 0,341 | 0,217 | 0,300 | 0,095 | 0,191 | 0,064 |
| Peptostreptococcus | 0,558 | 0,125 | 0,506 | 0,109 | 0,444 | 0,084 |
| Aggregatibacter | 0,260 | 0,106 | 0,254 | 0,102 | 0,200 | 0,077 |
| Parvimonas | 0,379 | 0,085 | 0,314 | 0,059 | 0,544 | 0,099 |
| Capnocytophaga | 0,302 | 0,116 | 0,356 | 0,135 | 0,224 | 0,045 |
| Eubacterium | 0,237 | 0,066 | 0,182 | 0,048 | 0,253 | 0,070 |
| Dialister | 0,134 | 0,041 | 0,084 | 0,033 | 0,096 | 0,035 |
| Corynebacterium | 0,089 | 0,027 | 0,108 | 0,049 | 0,264 | 0,078 |
| Atopobium | 0,565 | 0,136 | 0,577 | 0,157 | 0,567 | 0,135 |
| Treponema | 0,101 | 0,053 | 0,088 | 0,047 | 0,053 | 0,022 |
| Catonella | 0,042 | 0,015 | 0,028 | 0,009 | 0,022 | 0,008 |
| Lautropia | 0,039 | 0,018 | 0,058 | 0,022 | 0,090 | 0,026 |
| Alloprevotella | 0,574 | 0,170 | 0,432 | 0,160 | 0,298 | 0,100 |
| Filifactor | 0,107 | 0,042 | 0,064 | 0,031 | 0,043 | 0,017 |
| Kingella | 0,113 | 0,041 | 0,107 | 0,027 | 0,050 | 0,018 |
| Selenomonas | 0,263 | 0,117 | 0,125 | 0,037 | 0,123 | 0,046 |
| Mogibacterium | 0,144 | 0,041 | 0,125 | 0,037 | 0,206 | 0,044 |
| Bergeyella | 0,078 | 0,016 | 0,058 | 0,013 | 0,039 | 0,009 |
| Lactobacillus | 0,093 | 0,046 | 0,051 | 0,028 | 0,041 | 0,014 |
| Oribacterium | 0,332 | 0,060 | 0,306 | 0,066 | 0,306 | 0,070 |
| Campylobacter | 0,412 | 0,080 | 0,279 | 0,067 | 0,234 | 0,034 |
| Staphylococcus | 0,008 | 0,008 | 0,007 | 0,007 | 0,000 | 0,000 |
| Mycoplasma | 0,030 | 0,014 | 0,023 | 0,013 | 0,014 | 0,010 |
| Leptotrichia | 0,230 | 0,108 | 0,181 | 0,060 | 0,270 | 0,083 |
| Cardiobacterium | 0,016 | 0,006 | 0,013 | 0,006 | 0,004 | 0,002 |
| Scardovia | 0,015 | 0,007 | 0,008 | 0,006 | 0,000 | 0,000 |
| Bulleidia | 0,141 | 0,022 | 0,112 | 0,017 | 0,209 | 0,046 |
| Shuttleworthia | 0,006 | 0,005 | 0,003 | 0,002 | 0,003 | 0,002 |
| Actinobacillus | 0,143 | 0,060 | 0,254 | 0,106 | 0,185 | 0,120 |
| Eggerthia | 0,004 | 0,004 | 0,003 | 0,002 | 0,010 | 0,007 |
| [Prevotella] | 0,056 | 0,035 | 0,054 | 0,027 | 0,021 | 0,011 |
| Simonsiella | 0,006 | 0,004 | 0,022 | 0,011 | 0,010 | 0,010 |
| Mannheimia | 0,136 | 0,032 | 0,155 | 0,028 | 0,147 | 0,029 |
| Lachnoanaerobaculum | 0,123 | 0,028 | 0,095 | 0,030 | 0,106 | 0,024 |
| Olsenella | 0,004 | 0,002 | 0,004 | 0,003 | 0,004 | 0,003 |
| Actinobaculum | 0,002 | 0,002 | 0,002 | 0,001 | 0,001 | 0,001 |
| Propionibacterium | 0,006 | 0,003 | 0,004 | 0,002 | 0,008 | 0,004 |
| Alysiella | 0,001 | 0,001 | 0,000 | 0,000 | 0,000 | 0,000 |
| Eikenella | 0,014 | 0,010 | 0,013 | 0,009 | 0,014 | 0,012 |
| Peptococcus | 0,001 | 0,001 | 0,003 | 0,002 | 0,001 | 0,001 |
| Anaeroglobus | 0,003 | 0,002 | 0,000 | 0,000 | 0,000 | 0,000 |
| Brachymonas | 0,000 | 0,000 | 0,000 | 0,000 | 0,000 | 0,000 |
| Soonwooa | 0,000 | 0,000 | 0,003 | 0,003 | 0,000 | 0,000 |
| Solobacterium | 0,018 | 0,007 | 0,019 | 0,007 | 0,024 | 0,008 |
| Brevundimonas | 0,000 | 0,000 | 0,001 | 0,001 | 0,000 | 0,000 |
| Stomatobaculum | 0,084 | 0,019 | 0,073 | 0,019 | 0,209 | 0,073 |
| Fretibacterium | 0,005 | 0,005 | 0,000 | 0,000 | 0,001 | 0,001 |
| Nocardioides | 0,000 | 0,000 | 0,002 | 0,002 | 0,000 | 0,000 |
| Moryella | 0,000 | 0,000 | 0,000 | 0,000 | 0,001 | 0,001 |
| Clostridium | 0,038 | 0,016 | 0,029 | 0,013 | 0,067 | 0,023 |
| Alloscardovia | 0,003 | 0,003 | 0,009 | 0,006 | 0,000 | 0,000 |
| Slackia | 0,000 | 0,000 | 0,001 | 0,001 | 0,000 | 0,000 |
| Streptobacillus | 0,050 | 0,032 | 0,055 | 0,034 | 0,000 | 0,000 |
| Bacteroides | 0,000 | 0,000 | 0,000 | 0,000 | 0,005 | 0,004 |
| Chryseobacterium | 0,000 | 0,000 | 0,000 | 0,000 | 0,000 | 0,000 |
| Moraxella | 0,044 | 0,044 | 0,008 | 0,008 | 0,000 | 0,000 |
| Tannerella | 0,018 | 0,012 | 0,018 | 0,007 | 0,034 | 0,015 |
| Megasphaera | 0,250 | 0,071 | 0,119 | 0,041 | 0,224 | 0,061 |
| Butyrivibrio | 0,011 | 0,006 | 0,015 | 0,008 | 0,045 | 0,016 |
| Bifidobacterium | 0,032 | 0,026 | 0,081 | 0,077 | 0,015 | 0,013 |
| Lachnoclostridium | 0,003 | 0,002 | 0,000 | 0,000 | 0,001 | 0,001 |
| Mobiluncus | 0,000 | 0,000 | 0,000 | 0,000 | 0,000 | 0,000 |
| Metascardovia | 0,002 | 0,002 | 0,000 | 0,000 | 0,000 | 0,000 |
| Serratia | 0,204 | 0,057 | 0,271 | 0,062 | 0,162 | 0,048 |
| Cloacibacterium | 0,001 | 0,001 | 0,001 | 0,001 | 0,000 | 0,000 |
