# Supplementary material for: Analysis of the oral microbiome during hormonal cycle and its alterations in menopausal women: the “AMICA” project
Source: Sci Rep. 2022 Dec 21;12:22086. doi: 10.1038/s41598-022-26528-w (PMC9772230; doi:10.1038/s41598-022-26528-w)
Supplement: Supplementary file 13 — Supplementary Information 13. [file 41598_2022_26528_MOESM13_ESM.docx]

**Supplementary Table S5.**

| KEGG id | Entry type | KEGG name | p score |
| --- | --- | --- | --- |
| hsa00020 | pathway | Citrate cycle (TCA cycle) | 0,000263 |
| hsa00052 | pathway | Galactose metabolism | 0,007250 |
| hsa00280 | pathway | Valine, leucine and isoleucine degradation | 0,000001 |
| hsa00330 | pathway | Arginine and proline metabolism | 0,019718 |
| hsa00430 | pathway | Taurine and hypotaurine metabolism | 0,000537 |
| hsa00604 | pathway | Glycosphingolipid biosynthesis - ganglio series | 0,012562 |
| hsa04142 | pathway | Lysosome | 0,000857 |
| hsa04742 | pathway | Taste transduction | 0,014357 |
| hsa04922 | pathway | Glucagon signaling pathway | 0,035629 |
| hsa04940 | pathway | Type I diabetes mellitus | 0,049329 |
| hsa04973 | pathway | Carbohydrate digestion and absorption | 0,000118 |
| hsa04974 | pathway | Protein digestion and absorption | 0,000001 |
| hsa05230 | pathway | Central carbon metabolism in cancer | 0,000294 |
| M00009 | module | Citrate cycle (TCA cycle, Krebs cycle) | 0,008995 |
| M00010 | module | Citrate cycle, first carbon oxidation, oxaloacetate => 2-oxoglutarate | 0,000001 |
| M00133 | module | Polyamine biosynthesis, arginine => agmatine => putrescine => spermidine | 0,000376 |
| M00134 | module | Polyamine biosynthesis, arginine => ornithine => putrescine | 0,000001 |
| M00135 | module | GABA biosynthesis, eukaryotes, putrescine => GABA | 0,008492 |
| M00173 | module | Reductive citrate cycle (Arnon-Buchanan cycle) | 0,000285 |
| M00898 | module | Thiamine biosynthesis, pyridoxal-5P => TMP/thiamine/TPP | 0,031580 |
| 1.1.1.21 | enzyme | aldose reductase | 0,036723 |
| 1.4.3.22 | enzyme | diamine oxidase | 0,008868 |
| 1.5.3.13 | enzyme | N1-acetylpolyamine oxidase | 0,000001 |
| 2.1.4.1 | enzyme | glycine amidinotransferase | 0,011503 |
| 2.3.1.65 | enzyme | bile acid-CoA:amino acid N-acyltransferase | 0,000137 |
| 2.3.3.1 | enzyme | citrate (Si)-synthase | 0,000001 |
| 2.3.3.8 | enzyme | ATP citrate synthase | 0,000001 |
| 2.5.1.16 | enzyme | spermidine synthase | 0,037188 |
| 3.2.1.108 | enzyme | lactase | 0,001667 |
| 3.2.1.22 | enzyme | alpha-galactosidase | 0,000001 |
| 3.2.1.23 | enzyme | beta-galactosidase | 0,000001 |
| 3.2.1.46 | enzyme | galactosylceramidase | 0,000001 |
| 3.2.1.62 | enzyme | glycosylceramidase | 0,001680 |
| 3.4.13.18 | enzyme | cytosol nonspecific dipeptidase | 0,000001 |
| 3.4.13.20 | enzyme | beta-Ala-His dipeptidase | 0,001361 |
| 3.5.3.11 | enzyme | agmatinase | 0,000001 |
| 4.1.1.17 | enzyme | ornithine decarboxylase | 0,000001 |
| 4.1.1.22 | enzyme | histidine decarboxylase | 0,000001 |
| 4.2.1.3 | enzyme | aconitate hydratase | 0,000001 |
| 4.3.1.3 | enzyme | histidine ammonia-lyase | 0,000001 |
| 5.1.3.3 | enzyme | aldose 1-epimerase | 0,034975 |
| 6.2.1.2 | enzyme | medium-chain acyl-CoA ligase | 0,000001 |
| 6.3.1.17 | enzyme | beta-citrylglutamate synthase | 0,015831 |
| 6.3.2.11 | enzyme | carnosine synthase | 0,000794 |
| R00351 | reaction | acetyl-CoA:oxaloacetate C-acetyltransferase (... | 0,000001 |
| R00352 | reaction | acetyl-CoA:oxaloacetate C-acetyltransferase [... | 0,000001 |
| R00670 | reaction | L-ornithine carboxy-lyase (putrescine-forming... | 0,000001 |
| R01094 | reaction | D-galactose:NAD+ 1-oxidoreductase | 0,002457 |
| R01096 | reaction | D-galactose:NADP+ 1-oxidoreductase | 0,002457 |
| R01097 | reaction | D-Galactose:NADP+ 1-oxidoreductase | 0,002457 |
| R01101 | reaction | melibiose galactohydrolase | 0,001198 |
| R01104 | reaction | Galactosylglycerol galactohydrolase | 0,032868 |
| R01105 | reaction | Galactan galactohydrolase | 0,030809 |
| R01151 | reaction | Putrescine:oxygen oxidoreductase (deaminating... | 0,000001 |
| R01152 | reaction | N-Carbamoylputrescine amidohydrolase | 0,002674 |
| R01153 | reaction | S-Adenosyl-L-methionine:putrescine N-methyltr... | 0,002677 |
| R01155 | reaction | putrescine:2-oxoglutarate aminotransferase | 0,000001 |
| R01157 | reaction | Agmatine amidinohydrolase | 0,000001 |
| R01158 | reaction | L-histidinol:NAD+ oxidoreductase | 0,000001 |
| R01159 | reaction | S-adenosyl-L-methionine:L-histidine N-methylt... | 0,000001 |
| R01161 | reaction | L-histidine:2-oxoglutarate aminotransferase | 0,000001 |
| R01163 | reaction | L-histidinal:NAD+ oxidoreductase | 0,000289 |
| R01164 | reaction | L-histidine:beta-alanine ligase (ADP-forming) | 0,000001 |
| R01166 | reaction | Nalpha-(beta-alanyl)-L-histidine hydrolase | 0,000001 |
| R01167 | reaction | L-histidine carboxy-lyase (histamine-forming) | 0,000001 |
| R01168 | reaction | L-histidine ammonia-lyase (urocanate-forming) | 0,000001 |
| R01169 | reaction | S-adenosyl-L-methionine:L-histidine Nalpha-me... | 0,000001 |
| R01176 | reaction | Butanoate:CoA ligase (AMP-forming) | 0,000001 |
| R01179 | reaction | butanoyl-CoA:acetate CoA-transferase | 0,000001 |
| R01194 | reaction | 3-O-alpha-D-Galactosyl-1D-myo-inositol galact... | 0,000174 |
| R01324 | reaction | citrate hydroxymutase | 0,000001 |
| R01325 | reaction | citrate hydro-lyase (cis-aconitate-forming) | 0,000001 |
| R01365 | reaction | Butanoyl-CoA:acetoacetate CoA-transferase | 0,000001 |
| R01678 | reaction | Lactose galactohydrolase | 0,001580 |
| R01681 | reaction | hypotaurine:NAD+ oxidoreductase | 0,000001 |
| R01682 | reaction | 3-Sulfo-L-alanine carboxy-lyase (taurine-form... | 0,000001 |
| R01684 | reaction | taurine:2-oxoglutarate aminotransferase | 0,000001 |
| R01685 | reaction | taurine:ferricytochrome-c oxidoreductase (dea... | 0,000001 |
| R01688 | reaction | ATP:butanoate 1-phosphotransferase | 0,000001 |
| R01920 | reaction | S-adenosylmethioninamine:putrescine 3-aminopr... | 0,000001 |
| R01991 | reaction | L-histidine:4-aminobutanoate ligase (ADP-form... | 0,000333 |
| R01992 | reaction | alpha-Aminobutyryl histidine hydrolase | 0,000332 |
| R03355 | reaction | beta-D-Galactosyl-1,4-beta-D-glucosylceramide... | 0,000950 |
| R03617 | reaction | D-galactosyl-N-acylsphingosine galactohydrola... | 0,000001 |
| R03634 | reaction | Stachyose + H2O <=> Raffinose + D-Galactose | 0,007621 |
| R04019 | reaction | Digalactosylceramide galactohydrolase | 0,005044 |
| R04470 | reaction | Digalactosyl-diacylglycerol galactohydrolase | 0,034695 |
| R05320 | reaction | Taurine, 2-oxoglutarate:O2 oxidoreductase (su... | 0,029757 |
| R05652 | reaction | taurine:pyruvate aminotransferase | 0,000001 |
| R05961 | reaction | H2O + Globotriaosylceramide <=> D-Galactose +... | 0,000001 |
| R06010 | reaction | GM1 + H2O <=> GM2 + D-Galactose | 0,000001 |
| R07414 | reaction | glutamate putrescine ligase | 0,002681 |
| R07807 | reaction | G01977 + H2O <=> G13073 + D-Galactose | 0,000001 |
| R08544 | reaction | D-galactose:NAD+ 1-oxidoreductase | 0,002457 |
| R08714 | reaction | putrescine:pyruvate aminotransferase | 0,000001 |
| R09077 | reaction | spermidine:oxygen oxidoreductase (3-aminoprop... | 0,000001 |
| R09079 | reaction | carboxyspermidine:NADP+ oxidoreductase | 0,003129 |
| R10090 | reaction | citrate:N6-acetyl-N6-hydroxy-L-lysine ligase ... | 0,000184 |
| R10619 | reaction | D-galactose 1-epimerase | 0,030795 |
| R10677 | reaction | citrate:L-glutamate ligase (ADP-forming) | 0,015817 |
| R10686 | reaction | Pyridoxal phosphate + L-Histidine <=> 4-Amino... | 0,000001 |
| R10759 | reaction | 3-Hydroxyisovaleryl-CoA <=> 3-Hydroxyisovaler... | 0,000001 |
| R12304 | reaction | D-ornithine carboxy-lyase | 0,000001 |
| R12354 | reaction | N5-Citryl-D-ornithine + Citrate + ATP <=> Sta... | 0,028366 |
| C00124 | compound | D-Galactose | 0,000001 |
| C00134 | compound | Putrescine | 0,000001 |
| C00135 | compound | L-Histidine | 0,000001 |
| C00158 | compound | Citrate | 0,000001 |
| C00245 | compound | Taurine | 0,000001 |
| C00246 | compound | Butanoic acid | 0,000001 |
| C20827 | compound | 3-Hydroxyisovalerate | 0,000001 |
