# Supplementary material for: Analysis of the oral microbiome during hormonal cycle and its alterations in menopausal women: the “AMICA” project
Source: Sci Rep. 2022 Dec 21;12:22086. doi: 10.1038/s41598-022-26528-w (PMC9772230; doi:10.1038/s41598-022-26528-w)
Supplement: Supplementary file 14 — Supplementary Information 14. [file 41598_2022_26528_MOESM14_ESM.docx]

**Supplementary Table S1.** Salivary hormone levels measured by ELISA assays.

**Supplementary Table S2.** Microbiome composition profiles generated from 16S rRNA sequencing in terms of families in human saliva.

**Supplementary Table S3.** Microbiome composition profiles generated from 16S rRNA sequencing in terms of genera in human saliva.

**Supplementary Table S4.** Raw counts for metataxonomic 16S microbiome profiling at the genera rank.

**Supplementary Table S5.** Enrichment analysis on selected and more representative metabolites found in fertile/menopause class separation using the ‘PageRank ‘computational method showing pathways, modules, enzymes, reactions and metabolites involved with a threshold of *p*<0.001.

**Supplementary Figure S1.** Multivariate analysis of taxa at family level. **(A)** Scores plot of saliva samples collected from fertile subjects at their 3-rd (purple squares) and 14-th day (red squares) of the menstrual cycle and menopause women (green squares). **(B)** Loadings plot showing microbiota content responsible for saliva distribution in the model.

**Supplementary Figure S2.** Boxplot representing the species relative abundance of statistically significant taxa before FDR correction. Statistical analysis was performed using a linear model calculated on rank-corrected species relative abundances that included the experimental group and the smoking status of participant. P-values are not statistically significant after FDR correction.

**Supplementary Figure S3.** Pearson’s correlation coefficient for hormone levels and GH activities. P values are shown in the table.

**Supplementary Figure S4.** Pearson’s correlation coefficient for hormone levels and metabolites. P values are shown in the table.

**Supplementary Figure S5.** Salivary metabolic profile multilevel PLS-DA of M group (3^rd^ and 14^th^ day). **(A)** Sample representation: in this plot the saliva collected from subjects at their 3^rd^ (black dots) and the 14^th^ day (red dots) of the menstrual cycle are represented as points placed according to their projection in the smaller subspace spanned by the components (or latent variables) of our multivariate model. Confidence ellipses for each class are plotted to evaluate the strength of the discrimination (confidence level set to 95%). **(B)** Variables representation: NMR variables are represented through their projections onto the plane defined either by component1 and component2. The variables being assumed to be of unit variance, their projections are inside a circle of radius 1 centered at the origin called correlation circle. Strongly associated (or correlated) variables are projected in the same direction from the origin. The greater the distance from the origin the stronger the association. Two circumferences of radius 1 and 0.5 are plotted to reveal the correlation structure of the variables. In this analysis cut-off value was set to 0.5.

**Supplementary Figure S6.** Network plot showing pathways (red), modules (pink), enzymes (yellow), reactions (cyano) and metabolites (green) suggested by the enrichment analysis of discriminant metabolites based on Homo sapiens database in KEGG. Nodes are reported with a threshold of p<0.001.

**Supplementary Figure S7.** Pearson’s correlation coefficient for hormone levels and eCBs. P values are shown in the table.

**Supplementary Figure S8.** Influence of smoking on multiple factor analysis of saliva variables. Multiple factor analysis including variable groups meta-taxonomic analysis, glycoside hydrolases activities, NMR metabolomics and targeted lipidomics through mass spectrometry with estradiol levels, and anthropometric data show separation between experimental groups, and this separation remains after inclusion of smoking into the analysis.
